# Heterogeneity in sarcoma cell lines reveals enhanced motility of tetraploid versus diploid cells

**DOI:** 10.18632/oncotarget.14291

**Published:** 2016-12-27

**Authors:** Mohamed Jemaà, Samer Abdallah, Gwendaline Lledo, Gaelle Perrot, Tom Lesluyes, Catherine Teyssier, Pierre Roux, Juliette van Dijk, Frederic Chibon, Ariane Abrieu, Nathalie Morin

**Affiliations:** ^1^ Universités de Montpellier, 34293 Montpellier, France; ^2^ CRBM, CNRS, UMR 5237, 34293 Montpellier, France; ^3^ INSERM U1218, Bergonié Cancer Institute, F-33076 Bordeaux, France; ^4^ U1194 INSERM, IRCM, 34298 Montpellier, France; ^5^ Department of Pathology, Bergonié Cancer Institute, F-33076 Bordeaux, France

**Keywords:** sarcoma, CINSARC, mitosis, motility, diploid/tetraploid

## Abstract

Soft tissue sarcomas with complex genomics are very heterogeneous tumors lacking simple prognosis markers or targeted therapies. Overexpression of a subset of mitotic genes from a signature called CINSARC is of bad prognosis, but the significance of this signature remains elusive. Here we precisely measure the cell cycle and mitosis duration of sarcoma cell lines and we found that the mitotic gene products overexpression does not reflect variation in the time spent during mitosis or G2/M. We also found that the CINSARC cell lines, we studied, are composed of a mixture of aneuploid, diploid, and tetraploid cells that are highly motile *in vitro*. After sorting diploid and tetraploid cells, we showed that the tetraploid cell clones do not possess a proliferative advantage, but are strikingly more motile and invasive than their diploid counterparts. This is correlated with higher levels of mitotic proteins overexpression. Owing that mitotic proteins are almost systematically degraded at the end of mitosis, we propose that it is the abnormal activity of the mitotic proteins during interphase that boosts the sarcoma cells migratory properties by affecting their cytoskeleton. To test this hypothesis, we designed a screen for mitotic or cytoskeleton protein inhibitors affecting the sarcoma cell migration potential independently of cytotoxic activities. We found that inhibition of several mitotic kinases drastically impairs the CINSARC cell invasive and migratory properties. This finding could provide a handle by which to selectively inhibit the most invasive cells.

## INTRODUCTION

Soft tissue sarcomas (STS) [1] are rare mesenchymal malignant tumors that can derive from different cell lineage and from almost any soft tissue in the body [2]. The genome of STS cells may present simple translocations or harbor complex chromosome rearrangements that add another level of complexity to the lineage heterogeneity. As a result, the classification of STS, based on tumor cell differentiation and histological grading, is very challenging. Indeed, although these tumors account for less than 1% of human cancers, there are over 75 STS groups [3, 4]. Tumor resection followed by radiotherapy is widely used for low graded and localized tumors, but 5 years overall survival remains very poor. Patients that develop local relapse or distant metastasis, benefit of palliative chemotherapy treatments with a very low response rate [5].

Improved classification and identification of markers started with the development of genomic analyses and genetic characterization of STS. As a result, a list of specific gene mutations, amplifications and chromosome rearrangements is rapidly growing for many types of soft tissue sarcoma tumors [6]. Functional studies aimed at confirming altered pathways are developed with generation of sarcoma derived cell lines and *in vivo* models [7] and should allow the design of targeted therapies for patients developing STS tumors.

In this context, genomic and expression profiling of STS with highly rearranged genomes led to the identification of a validated 67 overexpressed genes signature called CINSARC for genome Complexity Index in SARComas. In STS and several other cancers, CINSARC predicts metastasis outcome with better confidence than the histological grading system developed by the French Federation of Cancer Centers Sarcoma Group (FNCLCC) [8, 9]. CINSARC is a molecular signature mostly composed of genes that encode cell cycle proteins controlling chromosome integrity and mitotic progression. Strikingly, most of the mitotic kinases and kinesins regulating the establishment and the evolution of the microtubule spindle during mitotic progression, required to faithfully segregate sister chromatids in daughter cells, are found among these 67 gene products.

Cell tetraploidization often occurs early in tumorigenesis [10] and may result from different insults to the cells such as telomere attrition [11, 12] or mitotic defects. For example, centriole amplification or overexpression of spindle assembly checkpoint proteins, but also cytokinesis failure or loss of AURKB dependent abscission checkpoint can all induce tetraploidization [12–20]. In normal cells, cytokinesis failure triggers activation of Hippo pathway, leading to TP53 dependent G1 arrest [1, 21–23]. However, when the *TP53*-dependent surveillance mechanism is lost, tetraploid cells will keep cycling, further promoting chromosome instability (CIN), aneuploidy and tumorigenesis [14, 18, 24]. To keep their proliferative advantage, derived cancer cells may adapt by inducing centrosomes clustering and/or silencing during mitosis [25–27].

Thus deregulation of mitotic genes observed in CINSARC positive tumors likely participates to the increasing complexity in chromosomes aberrations, rearrangements and instability observed in metastatic STS tumors.

Most intriguing, however, is the link that CINSARC signature makes between deregulation of mitosis and increased metastatic potential. As yet, only a few studies suggested that mitotic gene products could be linked to increased cell motility and invasive properties. Among them mitotic checkpoint proteins BUBR1 and Mad1 were proposed to regulate cellular motility through the regulation of MMP-2/9 metalloproteases and integrin secretion respectively [28, 29]. Most interestingly, identification of novel partners also directly linked mitotic CCNA2 and AURKA Kinase to the promotion of cell migration through RhoA and SSH1/CFL1 mediated regulation of actin dynamics [30, 31].

Here, we show that cell lines derived from CINSARC tumors acquired highly motile and invasive phenotypes. From these heterogeneous cell lines, we derived diploid and tetraploid clonal cell lines. RNA sequencing showed that clonal populations were positive for CINSARC signature and likely shared a same origin.

Relative to control fibroblast, overexpression of CINSARC transcripts was similar in all clones. In contrast, increase of most candidate mitotic gene products steady state levels was more elevated in tetraploid versus diploid cells. This did not confer significant proliferation advantage to tetraploid cells but strikingly increased their motility potential. In addition, we found that drug mediated inhibition of major mitotic kinases AURKA/B and TTK considerably reduced the migratory potential of all CINSARC clones.

## RESULTS

### CINSARC positive MFH137 and MFH152 sarcoma cell lines are hyper motile

**Figure 1:**
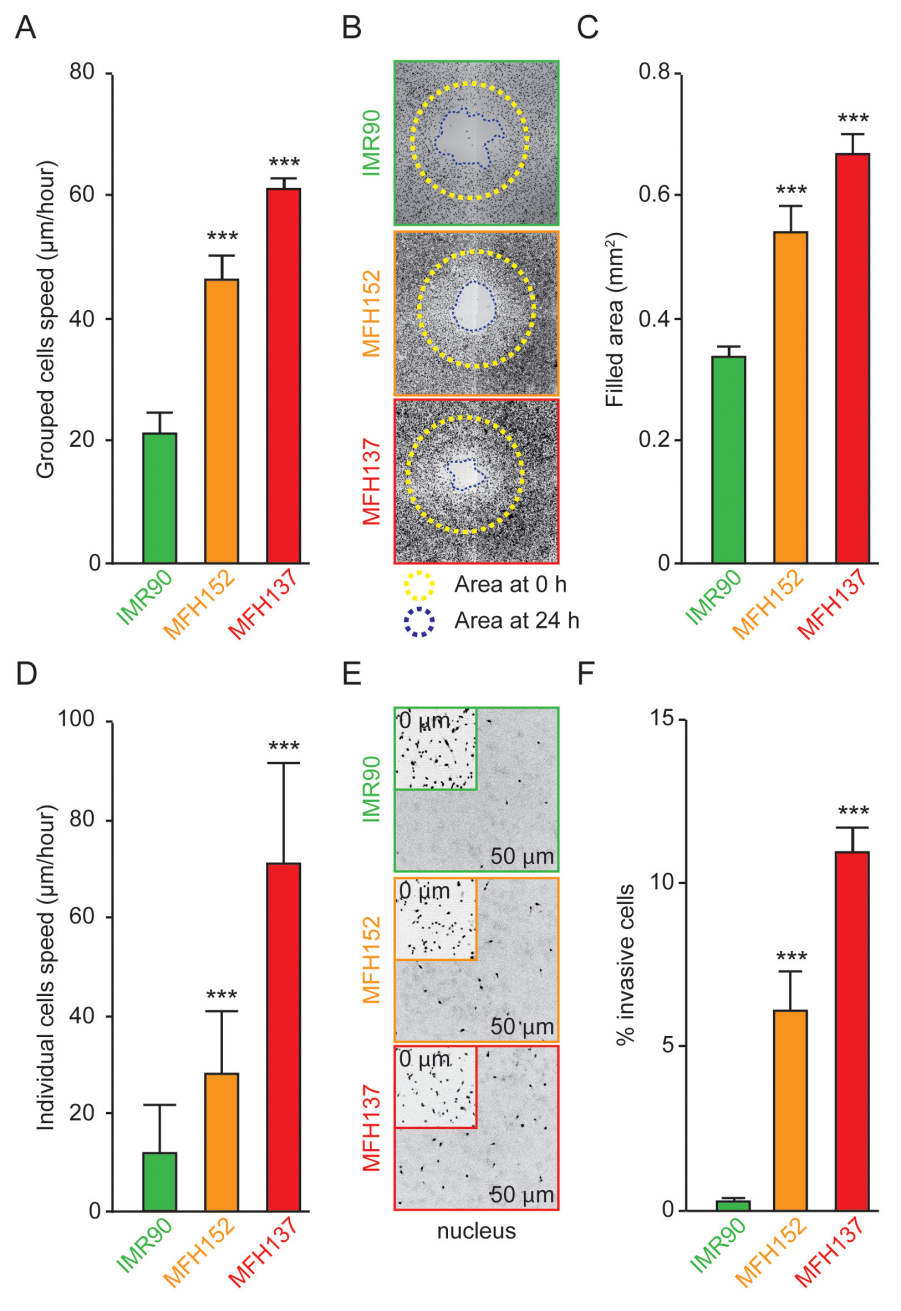
MFH137 and MFH152 sarcoma cell lines have a great invasive and motile potential **(A)** Confluent IMR90 fibroblast or MFH152 or MFH137 sarcoma cell monolayers were scratched with pipet tips and imaged every hour to study collective migration. Quantitative histogram shows the average speed, calculated from the wound closure rate, using image J software. **(B)** and **(C)**, two-dimensional migration assay using the Oris™ cell assay. Cells were allowed to migrate for 24h after the removal of cell seeding stoppers, fixed and stained with phalloidin to evaluate their motile potential. Representative photomicrographs are shown in panel **(B)**, the yellow broken lines show the empty migration zone at the start of the experiment while the blue lines delimit the cell-free area after 24h of migration. Quantitative data are presented in panel **(C)**. **(D)** Speed of individual IMR90, MFH152 and MFH137 cells was evaluated using the CytooChips Motility system to follow individual cell migration. **(E)** and **(F)** Invasive potential of IMR90, MFH152 and MFH137 cell lines measured after 24h. Representative micrographs show starting cell density at 0μm (**E**, insets) and invasive cells at 50 μm **(E)**. Quantitative data are represented in panel **(F)**. For all experiments, data are reported as means ± SEM (n ≥ 3). ***p < 0.001 (ANOVA test), as compared with the human fibroblast IMR90 cell lines.

We investigated the motile and invasive properties of CINSARC positive MFH152 and MFH137 sarcoma cell lines derived from metastatic tumors. Using a wound-healing assay to monitor cell migration, we found that MFH152 and MFH137 cells are extremely motile compared to non-transformed IMR90 fibroblast cells (Figure 1A and Supplementary Video 1). To avoid putative caveats from the wound procedure, we used size-controlled cell seeding stoppers from Oris™ and confirmed the high migratory potential of MFH137 and MFH152 cells (Figure 1B and 1C). Since these assays measure migration of grouped cells, we used fibronectin-coated narrow lines to monitor individual cells migration (Supplementary Video 2). As shown in Figure 1D, MFH137 and MFH152 still moved very fast, when forced to migrate individually. Finally, using a collagen layer-based 3D migration assay we found that MFH137 and MFH152 are highly invasive compared to untransformed fibroblasts (Figure 1E and 1F). In conclusion and as expected from the metastatic potential of the tumors they derive from, we found that MFH152 and MFH137 cells are highly motile and invasive *in vitro*.

### Cell cycle analysis reveals the heterogeneity of MFH152 and MFH137 sarcoma cell lines

**Figure 2:**
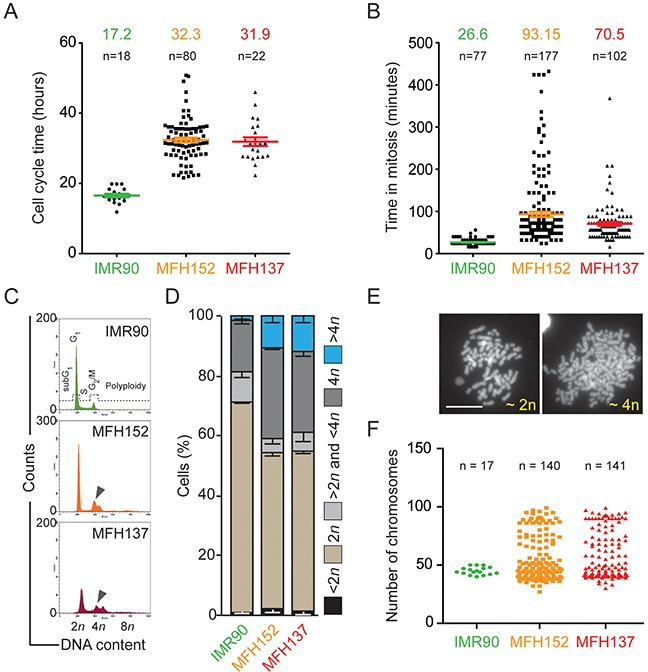
MFH137 and MFH152 sarcoma are a heterogeneous population **(A)** and **(B)** quantitative analyses of cell cycle and mitosis length of IMR90, MFH152 and MFH137 cell lines following image acquisition of the cell lines for up to 72h. Panel **(A)** displays the quantitative data of cell cycle duration, while panel **(B)** shows the length of mitosis. **(C)** and **(D)** IMR90, MFH152 and MFH137 cells lines were fixed and stained with propidium iodide for cytofluorometric assessment of cell cycle progression. Cell cycle distribution analysed by flow cytometry panel **(C)** and quantitative data (means ± SEM; n = 3) of corresponding flow cytometry profiles panel **(D)** are shown. **(E)** and **(F)** Metaphase spread of IMR90, MFH152 and MFH137 cells lines. Panel **(E)** shows examples of DAPI-stained chromosomes in the metaphase spreads with the corresponding ploidy while panel **(F)** reports quantitative data.

Since one striking feature of the MFH152 and MFH137 cells is to overexpress mRNAs involved in cell cycle regulation, we wondered whether it is correlated with cell cycle perturbations. To address this question, we constrained cells within fibronectin-coated circular areas and followed them by time-lapse microscopy over several divisions (72 hours, Supplementary Videos 3–5). The average cell cycle length was almost twice as long for MFH137 and MFH152 cells (32 hours) compared to IMR90 fibroblast cells (Figure 2A). In addition, cell cycle length was very heterogeneous between individual sarcoma cells lasting from 21 to 50 hours and from 22 to 46 hours respectively for MFH152 and MFH137 cells (Figure 2A). The cell heterogeneity was even more striking with mitosis duration that ranged from 24 min to 432 min (average 93 min) and from 16 to 368 minutes (average 70 min) for respectively MFH152 and MFH137 cells. By comparison, IMR90 spent 27 min average in mitosis (Figure 2B). Analyses of DNA content by flow cytometry showed that both MFH152 and MFH137 cell lines display an unusual proportion of cells containing at least 4n DNA content (Figure 2C–2D). To analyze whether the “shoulder” of the 4n DNA peak in MFH137 and MFH152 cells could represent G1 phase of a tetraploid subpopulation and/or aneuploid cell subpopulation, we performed metaphase chromosome spreads of these cells (Figure 2E). MFH152 and MFH137 cell lines contain three distinct cell populations with respectively 46, 92 or intermediate chromosomes number indicative of diploid, tetraploid and aneuploid cells (Figure 2F). Altogether our results suggest that the heterogeneity of the cell cycle in both sarcoma cell lines likely reflects the mixture of these cell lines subpopulations.

### Cell cycle profiles of diploid and tetraploid clones are similar but spindle assembly checkpoint is weakened in tetraploid clones

To be able to compare the behavior of diploid and tetraploid cells in the MFH137 and MFH152 sarcoma cell lines, we sorted them by flow cytometry using limiting dilution subcloning [32] and according to their size (Supplementary Figure 1A). A number of clones derived from single cells had a G1 profile with a 2n-4n intermediate content in DNA indicating strong chromosome instability (Supplementary Figure 1B). This aneuploid subpopulation was previously identified in the parental cell lines by metaphase chromosome spread (Figure 2F)

**Figure 3:**
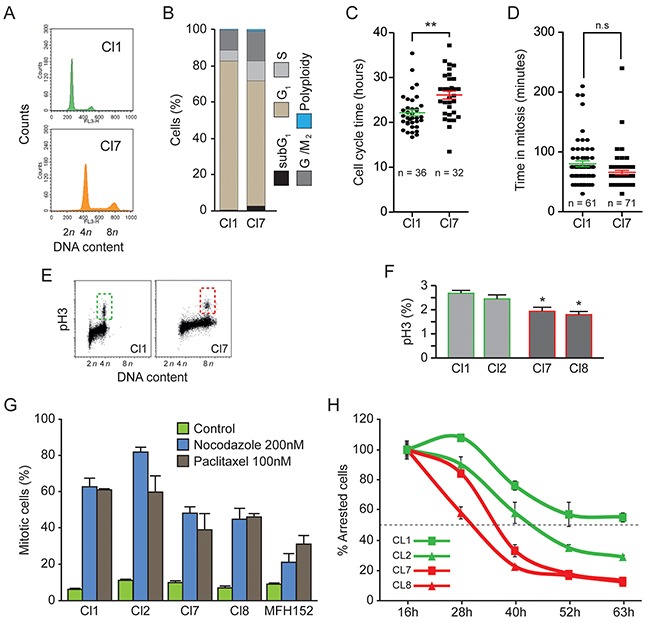
Cell cycle of diploid and tetraploid clones is similar but tetraploid clones have a weak spindle assembly checkpoint **(A)** and **(B)** Diploid (Cl1) and tetraploid (Cl7) MFH152 clones were fixed and stained with propidium iodide for the cytofluorometric assessment of cell cycle progression. Cell cycle distribution analysed by flow cytometry is displayed in panel **(A)**. Quantitative data of corresponding flow cytometry profiles are plotted in panel **(B)**. **(C)** and **(D)** Diploid (Cl1) and tetraploid (Cl7) MFH152 clones were imaged for up to 72h, by time lapse microscopy, to evaluate their respective cell cycle length and the time spent in mitosis. Panel **(C)** displays the quantitative data of cell cycle duration, while panel **(D)** shows the length of mitosis. **p < 0.05 (ANOVA test), n.s., not significant. **(E–F)** Fraction of G2/M cells present in two diploid and two tetraploid clones were analyzed by Flow cytometry profile **(E)** and quantified **(F)** following staining with antibodies directed against Ser10 Phospho-histone H3 (means ± SEM; n = 3). **(G–H)** Robustness of SAC in two diploid and two tetraploid clones. Percentage of the cell population arrested in mitosis following 16 hours exposure to carrier (control), nocodazole (200nM) or paclitaxel (100nM) **(G)** and percentage of the total number of arrested mitotic cells at 16 hours (100%) sustaining SAC over prolonged exposure to nocodazole (means ± SEM; n = 3).

We chose to further analyze diploid and tetraploid clones that had DNA content profiles, in agreement with respective 2n/4n and 4n/8n populations containing less than 20% of cells in G2/M phase (Supplementary Figure 1B, Figure 3A–3B for MFH152, Supplementary Figure 2A-2B for MFH137).

Flow cytometry analyses at different cell passages revealed that the DNA content of some of these clones remained stable for three months (data not shown). We thus characterized their cell cycle and motility profiles at early passages. A more homogenous cell cycle length was observed in subclones compared to parental cell line and averaged 22/26 hours respectively for diploid/tetraploid clones derived from MFH152 (Figure 3C). A similar behavior was observed for MFH137 cells (Supplementary Figure 2C). The statistically significant small variation of cell cycle length between diploid and tetraploid cells could reflect a required lengthening of G1 to G2 phases to prepare larger cells for cell division.

Duration of mitosis was similar in diploid and tetraploid clones derived from MFH137 and MFH152 cell lines (Figure 3D and Supplementary Figure 2D). To better compare the G2/M cell populations in diploid and tetraploid clones derived from MFH152 cells, we next analyzed by flow cytometry, their Ser10 Phospho-histone H3 content (Figure 3E). Quantification of the flow cytometry profiles shows that the fraction of Ser10 Phospho-histone H3 positive cells is largely similar in tetraploid and diploid clones (Figure 3F).

Cell tetraploidization is often linked to the weakening of the mitotic spindle assembly checkpoint (SAC). We thus investigated the strength of SAC in diploid and tetraploid clones derived from MFH152 cells. Nocodazole and taxol drugs interfere with microtubule dynamic and induce, in exposed cells, activation of the SAC and prometaphase arrest. Over a large range of drug concentrations, parental MFH152 cell line could not efficiently be synchronized likely reflecting the lack of SAC in the aneuploid cell subpopulation (data not shown). After 16 hours nocodazole or taxol drug exposure, diploid clones were more efficiently arrested (60-80%) than tetraploid clones (40-50%) and parental MFH152 cells (20-30%) suggesting that diploid cells respond better than tetraploid cells to checkpoint conditions (Figure 3G).

Next, to analyze, how spindle assembly checkpoint is sustained in arrested cells. The decay of all nocodazole arrested mitotic cells measured after 16 hours exposure (Figure 3G) was monitored over longer time of drug exposure. As observed (Figure 3H), tetraploid cells escaped faster (50% escape at respectively 30 and 36 hours for clone 8 and 7) than diploid cells (50% escape at respectively 46 and 63 hours for clone 2 and1) confirming the weakness of the SAC in tetraploid clones.

We conclude that the dispersion, we observed for mitosis length and cell cycle duration in parental cell lines, likely reflected the behavior of their aneuploid cell subpopulations. In contrast, MFH152 derived tetraploid and diploid clones have homogeneous and comparable cell cycle profiles with similar cell fraction in G2/M phases. Although the spindle assembly checkpoint is more robust in diploid than in tetraploid clones, the latter do not have significant proliferative advantage over the diploid cells.

### Sorting of diploid and tetraploid clones reveals that the tetraploid cells are more motile than their diploid counterparts

**Figure 4:**
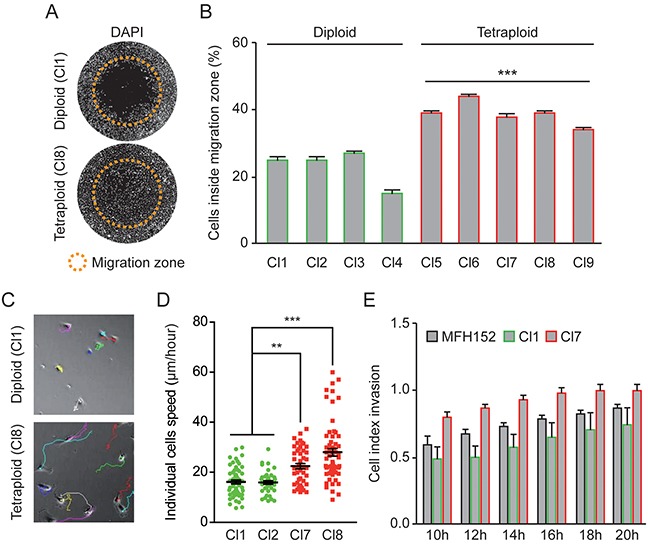
Tetraploid clones are more motile and invasive than diploid clones **(A–B)** Four diploid (Cl1-4) and five tetraploid (Cl5-9) MFH152 clones were plated using the Oris™ cell migration assay and grown for 24h before fixation. DAPI staining was used to evaluate the cells migratory potential. Representative micrographs are shown in panel **(A)** while panel **(B)** shows the percentage of nuclei inside the migration zone (means ± SEM; n = 3), ***p < 0.001 (ANOVA test, each tetraploid over each diploid). In panel **(A)**, orange circles show the migration zone at the start of the experiment (0h). **(C–D)** Two diploid (Cl 1-2) and two tetraploid (Cl 7-8) MFH152 clones were grown in non-confluent conditions and imaged for 24 hours, using time-lapse microscopy, to evaluate respective migration potential of individual cells. Panel **(C)** shows representative micrographs of the trajectories reconstituted using imageJ software of some cells from diploid (cl1) and tetraploid (cl8) clones, while panel **(D)** shows the individual cells speed (means ± SEM; n = 3), ***p < 0.001, **p<0.05 (ANOVA test). **(E)** Invasive behavior of cells from a diploid (Cl 1), a tetraploid (Cl 7) clones and MFH152 mother cell line was followed between 10 and 20 hours after seeding using the *xCELLigence* RTCA DP instrument (Ozyme, France) (means ± SEM; n = 4).

We next compared motile properties of diploid and tetraploid cells and found that tetraploid MFH152 cells migrate more efficiently than diploid cells (Figure 4A-4D).

First, motility assays were performed with confluent cells using cells seeding stoppers, as in Figure 1B. To avoid artifactual quantification related to diploid and tetraploid cell size difference, we counted the number of cells that penetrated inside the migration zone after 24 hours and found that roughly twice as many tetraploid cells colonized the empty space compared to diploid cells (Figure 4A-4B). The same assays performed on 2n and 4n MFH137 clones (Supplementary Figure 2E-2F) confirmed that motility is greatly enhanced in tetraploid compared to diploid sarcoma subclones.

Migrating cells may behave differently during collective cell migration, when cell/cell junctions play important functions, or as individualized cells. We therefore followed individual cells motility, of diploid and tetraploid clones derived from MFH152 cells, over a 20 hours time range. As for collective cell migration, we found that individualized tetraploid cells were significantly more motile than diploid cells (Figure 4C–4D).

We next sought to investigate and compare the invasive properties of MFH152 derived diploid and tetraploid clones. Cell Index Invasion was determined between 10 and 20 hours after seeding the cells. We found that tetraploid/diploid clones respectively display the most/the less invasive behavior while the parental MFH152 cells have an intermediate invasive behavior (Figure 4E).

Altogether, our results show that compared to diploid, tetraploid clones developed a more aggressive behavior characterized by increased motility and invasiveness.

### Diploid and tetraploid subclones are closely related and overexpress mitotic gene products

**Figure 5:**
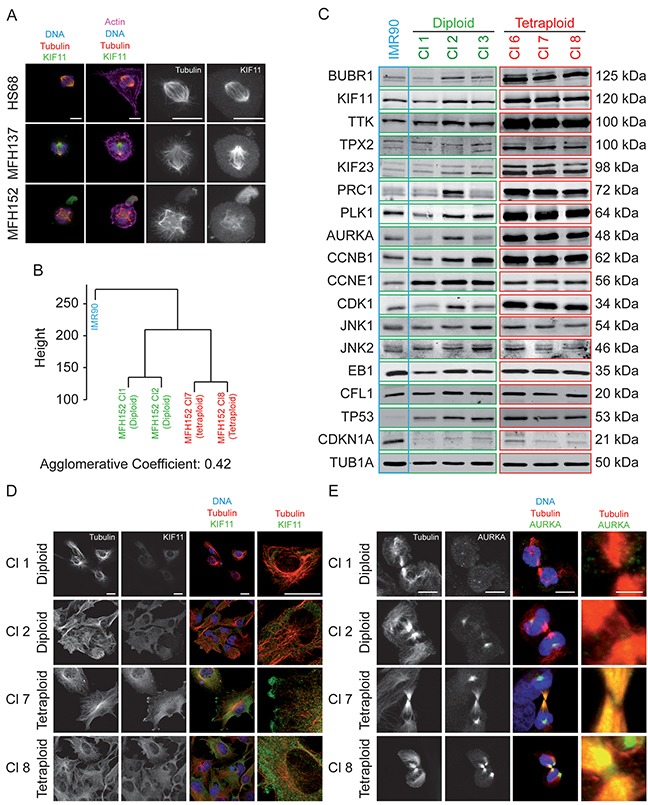
Tetraploid clones overexpress numerous proteins implicated in mitotic control and chromosome integrity **(A)** Metaphases of HS68 fibroblast, MFH137 and MFH152 parental sarcoma cells plated on L shape CYTOOchips™ coated with fibronectin and stained for indicated antibodies and for actin (phalloidin). Phalloidin staining shows that mitotic sarcoma cells have multiple blebs and do not adhere properly to the fibronectin coated L shape. Kinesin KIF11 is overexpressed and mislocalized in blebs. These mitotic figures are representative examples of the numerous mitotic defects observed in sarcoma cell lines (n>5). Detail of images of tubulin and KIF11 stainings are shown on the right. Bar is 10 μm. **(B)** Clustering results of IMR90 together with MFH152 diploid clones 1 and 2 together and tetraploid clones 7 and 8. **(C)** Three diploid (Cl 1-3) and three tetraploid (Cl 6-8) MFH152 clones (framed in green and red, respectively) and the IMR90 cell line (framed in blue) were collected for western-blot analysis of the indicated proteins involved in cytoskeleton, cell cycle and mitotic regulation. **(D–E)** Immunofluorescence of interphasic **(D)** or mitotic **(E)** cells from diploid (Cl 1-2) and tetraploid (Cl 7-8) MFH152 subclones were stained for tubulin together with KIF11 **(D)** or AURKA **(E)**. KIF11 is overexpressed in tetraploid clones. Detail of merge images between tubulin and KIF11 is shown on the right, Bars: 20 μm **(D)**. AURKA is overexpressed in midzone region of tetraploid clones. Detail of merge images between tubulin and AURKA is shown on the right, Bars: 15 μm **(E)**.

Many mitotic gene transcripts are deregulated in CINSARC positive cells [8] and abnormal mitotic figures are observed in these cells. As an example, strong overexpression of kinesin KIF11 is detected on metaphase spindle poles and in membrane blebs in MFH137 and MFH152 parental cell lines (Figure 5A).

Since diploid and tetraploid cell subclones derive from the same CINSARC positive tumor, we wondered whether their different motile behavior solely results from the specific properties of the cells they derive from inside the heterogeneous resected tumor.

To test this hypothesis, we performed transcriptome analyses of two diploid and two tetraploid MFH152 clones, together with IMR90 control fibroblast by RNA sequencing. Clustering results show that the two diploid clones together and the two tetraploid clones together were very similar with r=0.9901 and r=0.9939 respectively. A strong similarity was again observed between 2n and 4n clones (r=0.9701) while respectively more distant from the IMR90 (r=0.9114 and r=0.9046). In summary, transcriptomic profiles of 2n and 4n subclones are very closely related, as shown by the agglomerative coefficient that measures the clustering structure of the data set. Thus, they could be of same origin although this analysis does not definitely prove that they derive from each other (Figure 5B).

Since, mRNAs deregulation of CINSARC mitotic genes is conserved in derived diploid and tetraploid MFH152 subclones (Table 1 and Supplementary Figure 3) that are closely related but have different motility potential, we wondered whether representativity of mitotic gene products might be different in diploid and tetraploid subclones.

**Table 1:** CINSARC genes RNA Seq data for MFH152 diploid and tetraploid clones normalized to IMR90 expression levels

		MFH152			
	Diploid		Tetraploid	
GeneSymbol	IMR90	Cl 1	Cl 2	Cl 7	Cl 8
ANLN	1.0	3.0	2.9	3.1	3.2
ASPM	1.0	2.4	2.5	2.1	2.0
AURKA	1.0	1.8	2.1	1.6	1.5
AURKB	1.0	3.7	4.4	3.0	3.1
BIRC5	1.0	3.0	3.1	2.0	1.7
BUB1	1.0	3.6	3.2	2.9	3.0
BUB1B	1.0	1.8	2.2	1.7	1.7
CCNA2	1.0	1.5	1.8	1.2	1.0
CCNB1	1.0	1.9	2.4	1.8	1.3
CCNB2	1.0	2.2	3.0	2.3	1.9
CDC20	1.0	1.9	2.1	2.7	2.2
CDC6	1.0	1.7	1.6	1.5	1.7
CDC7	1.0	4.2	5.3	3.2	3.8
CDCA2	1.0	1.5	1.8	2.0	1.8
CDCA3	1.0	2.4	2.5	2.0	1.4
CDCA8	1.0	1.8	2.1	2.6	2.5
CENPA	1.0	2.9	3.5	2.4	1.8
CENPE/KIF10	1.0	2.2	2.2	1.2	1.0
CENPL	1.0	2.1	1.9	1.8	2.1
CEP55	1.0	2.9	2.6	2.2	2.0
CHEK1	1.0	1.4	1.5	1.3	0.8
CKS2	1.0	1.6	1.9	1.5	1.2
ECT2	1.0	2.6	2.9	2.7	2.3
ESPL1	1.0	2.3	2.4	2.4	2.3
FBXO5	1.0	2.3	2.5	2.5	2.4
FOXM1	1.0	2.7	3.2	2.8	2.2
H2AFX	1.0	1.7	2.0	1.2	0.8
HP1BP3	1.0	0.6	0.6	0.9	0.7
FANCI	1.0	3.0	3.4	4.2	4.6
Eg5/KIF11	1.0	2.2	2.1	1.9	1.7
KIF14	1.0	2.3	2.3	2.6	2.5
KIF15	1.0	1.6	2.0	1.6	1.7
KIF18A	1.0	2.7	2.8	1.7	1.9
KIF20A	1.0	1.0	1.4	1.5	1.1
KIF23	1.0	2.6	2.8	3.1	3.0
KIF2C	1.0	2.3	2.9	3.9	3.7
KIF4A	1.0	3.2	4.0	3.5	2.4
KIFC1	1.0	1.8	2.0	2.4	2.2
MAD2L1	1.0	2.0	2.2	1.4	1.2
MCM2	1.0	3.1	3.4	2.8	2.6
MCM7	1.0	2.7	2.7	3.0	3.0
MELK	1.0	3.1	3.7	3.2	3.2
NCAPH	1.0	4.6	3.5	4.2	4.8
NDE1	1.0	0.9	1.1	1.1	0.7
NEK2	1.0	2.6	3.2	2.8	2.3
NUF2	1.0	2.2	2.8	2.4	2.6
OIP5	1.0	3.9	4.4	3.5	2.8
PBK	1.0	2.4	2.9	3.3	3.1
PLK4	1.0	1.8	1.9	1.4	1.4
PRC1	1.0	2.0	2.1	2.2	1.9
PTTG1	1.0	6.4	7.5	7.3	5.1
RAD51AP1	1.0	3.4	3.7	3.3	3.6
RNASEH2A	1.0	2.9	4.5	3.1	3.0
RRM2	1.0	1.3	1.4	1.5	1.4
SGOL2	1.0	3.0	2.9	2.8	2.8
SMC2	1.0	2.0	2.0	1.9	2.1
SPAG5	1.0	1.9	2.0	1.4	1.5
SPC25	1.0	3.0	2.6	2.6	2.2
TOP2A	1.0	1.4	1.6	1.0	0.9
TPX2	1.0	4.2	4.5	4.0	3.5
TRIO	1.0	2.2	1.6	2.0	1.8
TRIP13	1.0	1.7	1.7	1.4	1.7
TTK	1.0	2.7	3.0	3.3	3.2
UBE2C	1.0	3.0	3.7	3.1	3.0
ZWINT	1.0	3.4	3.0	3.3	3.3

We analyzed, by western blot, the steady state level of several candidate mitotic and non-mitotic gene products in diploid and tetraploid MFH152 clones. We found that most mitotic proteins tested were only modestly overexpressed in diploid MFH152 clones compared to IMR90 cells. In contrast, overexpression of CCNB1, along with its associated kinase CDK1, other major mitotic kinases AURKA, BUBR1, PLK1, and TTK, the MAPs and kinesins PRC1, TPX2, KIF11, KIF23, were much increased in tetraploid than in diploid cell lines while non-CINSARC protein EB1, CFL1, JNK1, and JNK2 steady state levels were similar (Figure 5C). On the contrary, CCNE1, a G1/S gene product was mainly overexpressed in diploid cells. We also noted a strong overexpression of TP53 in both diploid and tetraploid clones. This is consistent with the fact that TP53 is mutated in MFH152 and MFH137 cell lines. Although stabilized, the mutant TP53 protein remains unable to promote CDKN1A expression, in these cells. In contrast to IMR90 fibroblast cells which have a low level of TP53 and high CDKN1A steady state level.

The differences in the steady state level of mitotic proteins, in diploid and tetraploid clones, unlikely results of cell cycle difference since diploid and tetraploid cells mitotic index are similar.

Interestingly, at the cellular level, we also found that kinesin KIF11 is more overexpressed in the cytoplasm of interphase tetraploid than in diploid MFH152 subclones (Figure 5D). Similarly, the interphase AURKA kinase centrosomal staining is more prominent in tetraploid than diploid cells (not shown), but more strikingly AURKA strongly stained the midzone and midbody region of mitotic tetraploid cells only (Figure 5E). Such a staining is similar to the one expected for Aurora B kinase and suggest that APC mediated proteolysis of AURKA may be partially defective in tetraploid clones.

Misexpression and overexpression of microtubule binding proteins together with regulatory kinases can result in profound microtubule and actin cytoskeleton rearrangements. Since actin and microtubule networks regulate the migratory capacities of cells, we asked whether overexpression of mitotic gene products confers to CINSARC positive cells their increased motility potential.

### Reversine and SP600125 inhibit sarcoma cell migration

To address this question, we developed a screen using 22 mitosis and cytoskeleton inhibitors at 3 different doses (0.1; 1 and 10 uM) for 24 hours (Supplementary Table 1). The initial screen was based on our assay of inhibition of cell migration using cell seeding stoppers. The first readout was the consequence of these treatments on cell death and the second was on cell migration. Cytotoxic effect inducing cell death was assessed by the ratio of counted nuclei (inside + outside migration zone) in the treated versus non-treated cells. We did not consider further, drugs that display a cytotoxic effect higher than 30% (Supplementary Table 2). Following the first round of analyses several drugs, with a too high cytotoxic effect, such as the PLK1 and CDK1 inhibitors, the CENPE and KIF11 kinesin inhibitors, the proteasome inhibitors and the cytoskeleton poisons were eliminated.

We then narrowed the screen to the drugs that passed the cytotoxicity test. We further tested the efficacy of these drugs at their non-toxic concentration to inhibit cell motility in chosen diploid and tetraploid MH152 subclones. We used the cell migration assay we developed, and counted the number of cells entering the migration zone. Table 2 summarizes the results, we obtained. We decided to consider 40% of migration inhibition, as a cut off, to consider the drug efficient in inhibiting the cell motility. Most of the kinesins, mitotic kinases and phosphatase inhibitors that proved to be very toxic at high doses, were only used at low concentrations with no noticeable effects on cell migration capacity (GSK 923295, Dimethylenastron, ZM 447439, RO3306, CDKi, Roscovitin, CDC25).

**Table 2:** Migration inhibition ratio for MFH152 diploid and tetraploid clones upon incubation with three doses of the indicated drugs

		Migration inhibition					
		Cell counts					
		0.1 μM		1 μM		10 μM	
		**Diploid**	**Tetraploid**	**Diploid**	**Tetraploid**	**Diploid**	**Tetraploid**
1	**AZ 3146**	0.00	0.00	0.12	0.00	N.R.	0.25
2	**Reversine**	0.04	0.00	0.08	0.00	0.40	0.47
3	**SP600125**	0.00	0.00	0.12	0.00	0.44	0.47
4	**ZM 447439**	0.00	0.10	N.R.	0.01	N.R.	N.R.
5	**Dimethylenastron**	0.00	0.03	N.R.	N.R.	N.R.	N.R.
6	**GSK 923295**	0.00	0.05	N.R.	N.R.	N.R.	N.R.
7	**SB203508**	0.00	0.03	0.00	0.01	0.01	0.12
8	**RO 3306**	0.18	0.00	0.01	0.00	N.R	N.R
9	**Cdk1 Inhibitor III**	0.00	0.05	0.00	0.00	N.R	N.R
10	**Roscovitine**	0.00	0.13	0.00	N.R	N.R	N.R
11	**NSC 95397**	0.12	0.08	0.15	0.11	N.R	N.R
12	**IPA3**	0.00	0.00	0.00	0.00	0.23	0.02
13	**Y-27632**	0.00	0.01	0.04	0.09	0.04	0.12
14	**ITX-3**	0.08	0.01	0.00	0.01	0.09	0.11
15	**Blebbistatin**	0.06	0.06	0.06	0.00	0.00	0.00

Green labeling indicates a migration inhibition ratio above 0.40.

Finally only the two drugs, Reversine and SP600125, met the criteria of conjugating low toxicity with significant inhibition of diploid and tetraploid sarcoma cells migration. These drugs target major mitotic kinases. Specificity of Reversine is best toward TTK (IC_50_ 6 nM) and good toward AURKB (IC_50_ of 98.5 nM) while SP600125 inhibits AURKA and AURKB (IC_50_ 60nM and 190nM) but also JNK (IC_50_ 100-200nM) and TTK (IC_50_ 1,9 μM).

**Figure 6:**
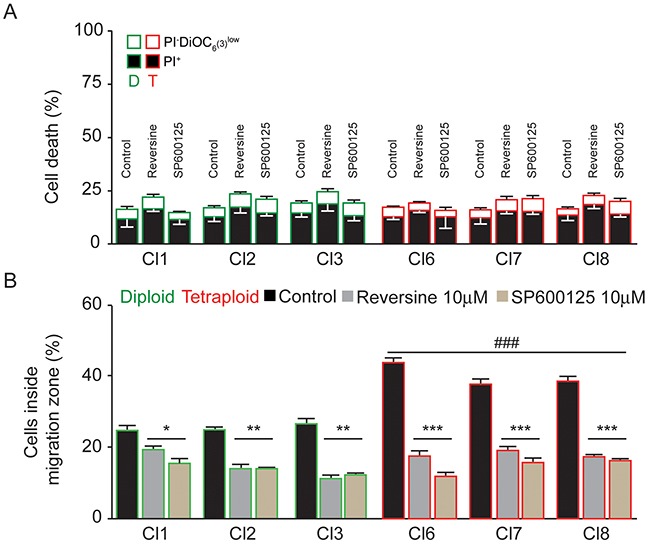
Reversine and SP600125 inhibit cell migration **(A)** Toxicity induced in diploid and tetraploid subclones following 24h treatment with either reversine or SP600125 was assessed by flow cytometry upon co-staining with the cell death–associated parameters dyes, propidium iodide (PI) and DiOC6 [72] (means ± SEM; n = 3). **(B)** Three diploid (Cl 1-3) and three tetraploid (Cl 6-8) MFH152 were plated using the Oris™ cell migration assay. Cells were grown with or without Reversine or SP600125 at 10 μM for 24h, fixed and stained with DAPI to measure their migratory potential. Quantitative data shows the percentage of cells (DAPI staining) inside the migration zone (means ± SEM; n = 3). *(p<0.05), **(p<0.01),***(p<0.001) indicates significant difference from the absence of both Reversine or SP600125 treatment while ### (p<0.001) indicates significant difference of the migration inhibition between all tetraploid clones taken one by one *vs* all diploid clones taken one by one (ANOVA test).

We focused our study on these promising two molecules, in order to better characterize their efficacy toward inhibition of migration. Assays were enlarged to 3 diploid sub-clones and 3 tetraploid sub-clones. We first and again evaluated the toxicity of the drugs using cytofluorometry-based assays. Measures of the cell death–associated parameters, vital dye propidium iodide (PI) and mitochondrial membrane potential (Δψm)-sensing dye DiOC_6_ [16]) confirmed the non toxic effect of both molecules at 24h on all sub-clones (Figure 6A). We next repeated the two-dimensional cell migration assays.

Both Reversine and SP600125 inhibited the migration of all sub-clones. Strikingly, the residual migration of the highly motile tetraploid subclones was similar to the diploid clones. These results show that tetraploid cells, that overexpress CINSARC mitotic gene products, were more sensitive to Reversine and SP600125 treatments (Figure 6B).

Next, to further study the effect of reversine and SP600125 on cell invasion, we used 3D MultiCellular Tumor Spheroid Assay (MCTS) that mimics several features of tumor microenvironment and privilege cell-cell adhesion over cell/extracellular matrix interactions [33]. Diploid cells efficiently formed regularly shaped spheroid after 24 hours culture. In contrast, even after 48-72 hours of culture, tetraploid cells only formed small cell clumps that broke easily and did not properly aggregate (data not shown), behaving as a number of tumor cell lines described in an extensive survey [33].

**Figure 7:**
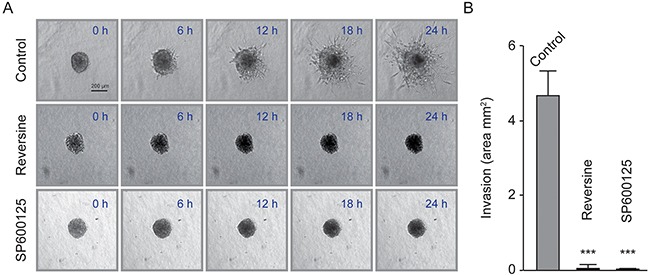
Reversine and SP600125 inhibit diploid MFH152 clone invasion in 3D **(A)** and **(B)** A diploid MFH152 clone was used to generate spheroids. Spheroids were transferred in collagen matrix (Panel **A**, 0 h) to allow invasion. Drug dilution buffer (Control) or Reversine or SP600125 were added to the collagen matrix and spheroids were imaged using time-lapse microscopy for up to 24 h to evaluate the drug effects on invasion. Panel **(A)** displays the fate of a single spheroid in the control and treatment conditions, at 0, 6, 12, 18 h while quantitative data of invasion are represented in panel **(B)**.

Since tetraploid MFH152 cells were unable to form spheroids, we concentrated on diploid MFH152 and initiated the 3D tumor invasion assay by transferring the spheroids in collagen-based medium and at low serum concentration (0.1%), in order to minimize cell proliferation. Using time-lapse imaging, we observed a remarkable induction of cell migration from the tight untreated MFH152 diploid spheroids embedded in the collagen matrix. Indeed, the spheroid volume rapidly expanded in collagen matrix, as invading cells that induced matrix proteolysis/degradation colonize the newly emptied space (Figure 7A and Supplementary Video 6). In contrast, when spheroids were incubated in collagen-based medium containing either reversine or SP600125, a complete inhibition of invasion was observed (Figure 7A and Supplementary Video 7–8) and quantified (Figure 7B).

This drug-mediated inhibition of invasion cannot be attributed to mitotic arrest since we did not observe any spheroids shape changes/expansion following drug treatment, even at very early time points (Supplementary Videos 6–8).

### The combination of several CINSARC mitotic gene products is likely required to upregulate tetraploid sarcoma cells migration

Reversine and SP600125 target several major kinases. In order to identify whether one of these kinases could specifically regulate sarcoma cells migration. We focused on AURKA, AURKB and TTK kinases that are targeted by reversine and SP600125 and also chose KIF11 that strongly stains the lamellipodia of interphase tetraploid cells (Figure 5D). We used siRNAs directed against AURKA, AURKB, TTK and KIF11 and highly specific drugs commercially available (Supplementary Table 2). MLN8237 (Alisertib), a selective AURKA inhibitor (IC50 of 1.2nM in cell free assay, with >200 fold higher selectivity for AURKA than AURKB); AZD1152-HQPA (Baraserib) that is highly selective against AURKB (IC 0.37nM is cell free assay, with >3700 fold more selective for AURKB than AURKA); SB743921, a KIF11 inhibitor (K_i_ of 0.1 nM, with almost no affinity to other kinesins); Mps-BAY2a, a TTK inhibitor (IC50 of 1nM).

Consequences of siRNAs and drug treatments on cell migration potential were tested with the cell seeding stoppers assay (Oris ™). Almost complete protein depletion was observed with 5nM siRNA treatment, none of the siRNAs displayed a cytoxic effect higher than 30% when used at 100nM (Supplementary Figure 4A). However, when tested at 5 and 100nM, none of the siRNA treatment induced significant effect on either diploid or tetraploid cell migration capacity (Supplementary Figure 4B).

To confirm these results, we used the specific inhibitors described above. First, diploid (clone 1) and tetraploid cells (Cl7) were exposed for 24 hours to a range of drug concentration to determine by flow cytometry their cell cycle profile (Supplementary Figure 5A-5B). Deregulation of AURKA kinase induces centrosomal abnormalities, defective chromosome segregation followed by aneuploidy and/or cell death. MLN8237 (AURKAi) inhibits AURKA at low nanomolar range (10nM). But, as reported [34], MLN8237 concentration above 100nM induced AURKB inhibition dependent cell polyploidization. KIF11 is required for bipolar spindle assembly and KIF11 defects result in monopolar spindle formation and mitotic arrest [35], a phenotype we observed with SB743921 concentrations, as low as 10nM. Cytokinesis defect and polyploidization resulting from AURKB dysregulation [36] was induced by 20nM AZD1152-HQPA treatment. We previously reported that Mps-BAY2a mediated TTK inhibition prevents SAC activation and ultimately results in aneuploidization/polyploidization [37]. Here, cell cycle perturbations were induced with Mps-BAY2a concentration as low as 100nM.

Cell cytotoxicity remained below 30% both for diploid and tetraploid clones using these defined low but effective inhibitor concentrations (Supplementary Figure 5C-5F). Nevertheless, when used at similar concentrations, none of these drugs had a significant inhibitory effect on either diploid or tetraploid cell motility (Supplementary Figure 5G-5H).

In summary, interference with chosen single mitotic gene products is not sufficient to inhibit cell migratory activity. We suggest that it is the combination of several deregulated mitotic gene products of CINSARC signature that is responsible for the increased migratory capacity of tetraploid clones. Inhibition of cell invasiveness and motility mediated by SP60025 and reversine that have broader effects on several mitotic gene products further support this hypothesis.

## DISCUSSION

Mitosis is a highly regulated process that leads, in most cells, to the equal partitioning of chromosomes and cytoplasmic material in the two daughter cells. In normal tissues, protective mechanisms insure that tetraploidization does not happen [1, 19, 38] and if an accident occurs the resulting tetraploid cells are rapidly eliminated [39, 40]. Nonetheless, cycling tetraploid cells with supernumerary centrosomes and aberrant mitoses are observed at very early stages of tumorigenesis [10, 41–43] and surviving tetraploid cells were proposed to trigger chromosome instability (CIN) and aneuploidy in absence of *TP53*-mediated G1 checkpoint [12, 18, 24, 44, 45]. Indeed, tetraploidy associates with increased DNA damage, abnormal bipolar/multipolar mitoses, chromosome breaks and/or missegregration [10, 12, 20, 46, 47].

But, in contrast to functions in tumorigenesis, few data exist concerning a potential involvement of tetraploidization in the promotion of cell migration and/or invasion [18].

In tumors, cells that become invasive must detach from other cells and degrade the extracellular matrix through which they will migrate. Invasive behavior requires profound changes of cell shape, adhesion and mechanical properties that depend upon dynamic changes of actin, microtubule but also intermediate filament networks [48–51].

In this study, we showed that cell lines derived from CINSARC positive soft tissue sarcoma have, as expected, highly motile profiles and enhanced capacities to migrate compared to non-transformed fibroblasts. Because of the heterogeneity of the cell lines derived from these tumors, we isolated cellular clones that proved to be aneuploid, for most. We were, however, capable to isolate and grow diploid and tetraploid clones that remain stable over a three months period. Unexpectedly, the motile and invasive behavior of tetraploid clones was greatly enhanced compared to diploid cell clones. RNA sequencing indicated that they likely share the same origin and kept the CINSARC signature with similar levels of deregulated mitotic gene transcripts. Nonetheless, the overexpression of candidate mitotic gene products, i.e. proteins, was greatly enhanced in the tetraploid population. Since mitotic indexes were similar in diploid and tetraploid cells, we assume that tetraploid dependent increased steady state level of mitotic proteins depends upon a regulation that is beyond the transcriptional level, and acquired with tetraploidization. Degradation of many mitotic proteins occurs at the end of mitosis and is required either to exit mitosis or to prevent inappropriate functioning in interphase [30, 52–58]. Although, we do not know how mitotic proteins are stabilized in tetraploids [59], it is tempting to speculate that ubiquitin-mediated proteasome degradation of proteins at the end of mitosis could be partly defective. Indeed, we observed by immunofluorescence that a number of mitotic proteins, such as KIF11, KIF23 and PLK1 were overexpressed in interphase in sarcoma cell lines (data not shown). KIF11 kinesin, which is overexpressed in tetraploid sarcoma cells, was shown to be involved in regulation of cell migration [16, 60]. In addition, AURKA accumulation, at the midzone and midbody region, in late mitosis of tetraploid sarcoma cells also indicates that its Apc/C-cdh1 mediated proteolysis is deficient in these cells. While AURKA degradation is important for the organization of the spindle during anaphase [61] [62], a link was also proposed between stabilization of AURKA and tumorigenesis [63].

Overexpression of mitotic gene products after the completion of mitosis would also be consistent with the fact that invading cancer cells are mostly in G0/G1 [64].

We identified that inhibitors targeting the major mitotic kinases AURKA/B and TTK inhibited cell migration of diploid and tetraploid sarcoma cell subclones and invasion from diploid spheroids. Thus, misexpression of these mitotic kinases regulates the motile behavior of sarcoma cells. However, using highly specific drugs and targeted siRNA approaches, we failed to pinpoint novel functions of a specific mitotic gene product in upregulating motility.

This further suggests that it is a combination of several deregulated mitotic gene products that participate to the cytoskeleton remodeling responsible for the increased migratory capacity observed in tetraploid clones. Inhibition of cell invasiveness and motility mediated by SP60025 and reversine that affect several mitotic gene products further support this hypothesis.

In the future, it will be of interest to study the motile behavior of *TP53*^-/-^ tetraploids derived from non-CINSARC diploid cells together with the diploid parental cells. This will allow to address whether tetraploidization by itself can promote increased cell motility and eventually whether such a phenotype will be linked to the stabilization of mitotic gene products.

Deregulation of many mitotic gene product observed in CINSARC signature correlates with high metastatic potential [8]. We show that the significance of CINSARC signature, over motile and invading cell behavior, derives from over/misexpression of mitotic kinesins and kinases. Our study further highlights novel functions of mitotic proteins in regulation of cell motility [28–31]. Validation of CINSARC signature in different tumor types [8, 65] increases its importance for prognosis and potential targeted therapies. In this context, it would be of interest to focus on the understanding of the origin of deregulation of so many mitotic gene transcripts. In that regard, an interesting candidate will be the FoxM1 transcription factor that regulates, as a bulk, most kinetochores genes [66] and which is highly expressed in sarcoma [67].

## MATERIALS AND METHODS

### Cell lines and culture conditions

MFH137 and MFH152 cell lines derive from two distinct tumors of mesenchymal origin that were initially characterized as malignant fibrous histiocytoma (MFH) and were subsequently reclassified as undifferentiated pleomorphic sarcoma (UPS). Isolation of MFH137 cell line was previously described [68]. Briefly, sterile fragment from resected tumor was minced in culture medium and then disaggregated by overnight incubation in collagenase (100 units/mL) at 37°C. Long-term culture (more than 70 passages were done) and standard harvesting procedures were used. We followed the same procedure to isolate the MFH152 cell line. The consistency between the cell lines and the original tumors was done by comparison of the genomic profiles.

IMR90 human fibroblast cell line was a gift of Dr. DA Skoufias (IBS, Grenoble). All cell lines were cultured in Dulbecco's modified Eagle's medium (DMEM) supplemented with 10% foetal calf serum and antibiotics.

### Reagents and antibodies

The drugs used for screening in this study are listed in Supplementary Table 1.

Reversine, SP600125, ZM 447439, BI 2536, STLC, Dimethylenastron, SB203508, RO 3306, Cdk1 Inhibitor III, Roscovitine, NSC 95397, IPA3, Y-27632, ITX-3, Nocodazole, Paclitaxel, Blebbistatin, Cytochalasin B, MG 132 and Velcade were purchased from Sigma–Aldrich (St. Louis, MO, USA). AZ 3146 and Mps-BAY2a were obtained from Tocris (Bristol, United Kingdom). MLN8237 (Alisertib), AZD1152-HQPA (Baraserib) and SB743921 were purchased from Selleckchem. GSK923295 is from MedChem express.

Monoclonal antibodies against α-tubulin, CDK1, AURKA, PLK1, KIF11 (Sigma-Aldrich); CCNB1, CCNE1, TP53, CDKN1A, JNK1, JNK2 (Cell signaling); EB1, COFILIN (Santa Cruz), TTK, BUBR1 (Abcam). Polyclonal antibodies against PRC1 (Santa Cruz), Tpx2 [69]; Kif23 [70] were used.

### Migration assays

For wound-healing assay, scratches were performed, with sterile 1000 μl tips, on confluent cell monolayers and monitored every hour by time-lapse microscopy. The two dimensional Oris cell migration assays were performed according to the manufacturer's instructions (Platypus Technologies, Madison, USA). Briefly, cells were seeded (2 × 10^4^ cells per well) into 96-well plates with “silicone stopper” and grown overnight. Then stoppers were removed and cells were incubated for an additional 24h to allow their migration into the empty zone. Cells were fixed with 4% PFA in PBS, stained with DAPI (for DNA detection) and FITC-phalloidin (for actin staining). Data acquisitions were performed using Cellomics Arrayscan (Arrayscan VTI Live; Carl Zeiss, Inc). The number of cells in the migration zone and the total number of cells outside and inside the migration zone (same area for both zones) were counted, by thresholding the DAPI stained nuclei, using image J software. The ratio between these two numbers was used as the percentage of migrating cells. For individual cell migration (Figure 1), cells were plated on glass coverslip coated with fibronectin on linear tracks of different widths (CYTOO™Chips Motility, CYTOO). Cells were seeded at low concentration (approximately 20 cells per mm^2^), to avoid cell-cell contacts that would affect analyses. For individual cell migration (Figure 4C–4D), cells were plated on 6 well plates at very low density (30000 cells per well). Cells were monitored by time-lapse microscopy. Image acquisitions were performed every 15 or 30 min (Figure 1D/Figure 4C–4D) for 24h using a Leica DMIRE2 inverted cell microscope equipped with a LMC 20 × 0.4 lens (Leica, Wetzlar, Germany). Time lapse were analyzed using ImageJ software (freely available from the National Institute of Health, http://rsb.info.nih.gov/ij/).

### Invasion assays

3D invasion assays (Figure 1E–1F) were performed in 96-well plates (PerkinElmer) coated with 0.2% BSA (Sigma-Aldrich) and 10^4^ red fluorescent polystyrene microspheres (FluoSpheres; Invitrogen). In brief, cells were suspended in 2.3 mg/ml serum-free liquid bovine collagen at 10^5^ cells/ml. 100 μl aliquots were dispensed into the plates. Plates were centrifuged at 1,000 rpm and further incubated at 37°C. Once collagen had polymerized, serum was added on top of the collagen to a final concentration of 5% (final volume of 30 μl). After 24 h, cells were fixed with 4% PFA and stained with Hoechst 33342 (Invitrogen). Images were acquired from each well at 50 and 0 μm from the bottom of the well using a Cellomics Arrayscan. The invasion index was calculated as the ratio of the cell number at 50 μm versus 0 μm.

Invasion experiments (Figure 4E) were performed with the *xCELLigence* RTCA DP instrument (Ozyme, France) according to the manufacturer's guidelines. 24 hours prior experiment, cells were deprived of fœtal calf serum. A layer of Matrigel (300μg/ml, BD Biosciences) was applied on the CIM-Plate 16 upper chamber membranes as described [71]. Subsequently, the coated upper chambers were incubated at 37°C to homogenously gelify during a minimum of four hours, followed by addition of 160 μL media to the lower and 30 μL serum free media to the upper chambers. 20 000 cells were seeded in every well of the upper chambers. Cell Index (CI) of each well was automatically monitored with the *xCELLigence* system every hour during a 24 hour period. Each condition was performed in quadruplicate. Cell Index Invasion represents the ratio of Cell Index of Matrigel-coated wells (invasion) to Cell Index of uncoated wells (migration) at specific time points.

In 3D MultiCellular Tumor Spheroids assay MCTS, spheroid formation was performed by incubating the cells (1000) in presence of 2.4% methylcellulose in U-shaped bottom wells of 96-wells plate. After 24hours, formation of multicellular spheroids was observed for diploid clones. Then, using 100 μl pipet tips diploid spheroids were transferred to wells of 96-wells plate and embedded into a collagen matrix for the invasion assay (Invasion matrix contains collagen (PureCol, Sigma) mixed with culture medium at 0.1% FCS with/without drugs). Spheroids behavior was monitored every hour by time-lapse microscopy. Z stack images that spanned the entire size of the spheroids were acquired at every time point. Relative cell invasion/expansion was quantified using ImageJ software. The area colonized by cells, away from the spheroids, was measured in several microscopic slices in the middle of the spheres and compared to the original spheroid diameter. The largest surface was arbitrarily chosen as the representative value of invasive potential for the specific condition.

### Measures of cell cycle length and mitosis duration

Cells were seeded on glass coverslip coated with adhesive discs of fibronectin (CYTOOchips™ Motility, CYTOO, France) and imaged every 15 minute for 72h with a Leica DMIRE2 inverted microscope with a LMC 20 × 0.4 lens and appropriate filters (Leica, Wetzlar, Germany). Images were analyzed with Image J software. Cell cycle length was estimated by measuring the time interval between the rounding of the same cell at mitotic entry. Length of mitosis was measured, as the time interval between cell rounding (mitotic entry) and start of spreading after mitotic exit.

### Cytofluorometric studies

- For the assessment of cell cycle distribution, cells were collected, washed once with 0.1% (w/v) D-glucose (Sigma-Aldrich) in PBS and then fixed by gentle vortexing in ice-cold 75% (v/v) ethanol for 30 sec. After overnight incubation at -20°C, samples were centrifuged, PBS washed and stained with 50 μg/mL PI in 0.1% (w/v) D-glucose in PBS supplemented with 1 μg/mL (w/v) RNase A (Sigma-Aldrich) for 30 min at 37°C. Afterwards, samples were incubated for at least 2 h at 4°C before cytofluorometric analysis.

- Flow cytometry acquisition and analyses to determine the plasma membrane integrity and mitochondrial transmembrane potential (Δψm) were performed as described previously [72]. Briefly, cells were collected and stained with 1 μg/ mL propidium iodide (PI), which only incorporates into dead cells, and 40 nM 3,3′-dihexyloxacarbocyanine iodide (DiOC_6_(3), a Δψm-sensitive dye for 30 min at 37°C before FACs acquisition.

- Cytofluorometric sorting of diploid and tetraploid cells was performed with a FACSAria cell sorter and the gating of small and big cell was based on the size and granularity parameters using the normal light scattering parameters Forward Scatter (FSC) *vs* Side Scatter (SSC). Single cells, sorted from mother cell line, were seeded in 96-well plates. 1000 “small” plus 1000 “big” cells for each MFH152 and MFH137 cell lines were sorted. After 15 days, surviving clones were cultured in 6-well plates. We succeeded to isolate stable tetraploid clones (3 from MFH137 and 5 from MFH152) and stable diploid clones (more than 20 for each cell line) for up to 3 month. The majority of surviving clones were aneuploid.

- To measure histone H3 phosphorylation on serine 10, cells were fixed with 75% (v/v) ethanol in PBS, permeabilized with 0.25% (v/v) Tween 20 in PBS and stained with a rabbit antiserum specific for phosphorylated histone H3 (rabbit polyclonal IgG1 #06–570, Millipore-Chemicon International), as previously described [73]. Cytofluorometric acquisitions were performed by means of a FACSCalibur (BD Biosciences).

### Measures of spindle assembly checkpoint robustness

A range of concentration of microtubule dynamic interfering drugs nocodazole and taxol was first assayed on sarcoma cells. Mitotic arrest, indicative of activation of the spindle assembly checkpoint, was quantified by counting the fraction of round cells in the total cell population, following 16 hours drug exposure using optimized drug concentration (200nM nocodazole and 100nM taxol). To evaluate SAC robustness, diploid (Cl1, Cl2), tetraploid (cl7, cl8) and MFH152 cells were seeded on six well plates, 200nM nocodazole was added after 2 hours after cell adhesion. Cells were imaged every hour for 64h with a Leica DMIRE2 inverted microscope with a LMC 20 × 0.4 lens and appropriate filters (Leica, Wetzlar, Germany). All mitotic cells at 16 hours were assigned a 100% for every condition. The loss of spindle assembly checkpoint, characterized by mitotic exit of these cells was determined at 28, 40, 52 and 63hours in presence of the drugs. Images were analysed with Image J software.

### Chromosome spreads

Cells were treated with 100nM nocodazole for 16h to enrich the percentage of mitotic cells, then collected and subjected to hypotonic lysis by incubation in 75mM KCl for 10 min at 37°C. After removal of hypotonic solution, cells were fixed in freshly prepared Carnoy solution (3/1 methanol/acetic acid) and stored at -20°C. Fixed cells were dropped onto pre-cooled glass microscope slides and dried at room temperature. Chromosomes were stained with 100ng/ml DAPI (Molecular Probes–Invitrogen) and mounted in Vectashield H-1000 mounting medium (Vector Laboratories, Burlingame, CA, USA). Fluorescent images were acquired using a Zeiss AxioimagerZ1 motorized microscope (Zeiss, Oberkochen, Germany) driven by MetaMorph (Molecular Devices).

### Immunofluorescence microscopy

Immunofluorescence was performed as described [69]. Briefly, cells were either fixed in methanol for 5min at −20°C or in 4% PFA (in PBS with 0.2% Triton X-100), immunostained with indicated primary antibodies Secondary antibodies conjugated to Alexa fluorochromes (Thermo Fisher Scientific-Invitrogen) were used. F-actin and DNA were, respectively, stained with phalloidin-Atto647 and DAPI (Sigma-Aldrich). For analyses of mitotic parental cells, cells were seeded on L shape CYTOOchips™, as recommended by the manufacturer. Confocal microscopy was performed using a Leica SP8-UV microscope equipped with Leica 63x HCX PL APO 1.4 oil CS2 objective. For quantitative comparison, fluorescent staining of respectively KIF11 or AURKA were acquired using same laser power and conditions. Stack images were acquired and maximum intensity projection (MIP) of images (keeping same number of grey levels between images) were analyzed with Image J software.

### RNA sequencing

Total RNA from IMR90 and from two diploid and two tetraploid MFH152 subclones were prepared with a TRIzol® Plus RNA Purification Kit (Thermofisher) according to the manufacturer's instructions. The library from total RNA was prepared using the TruSeq®Stranded Total Sample Preparation kit (Illumina Inc.) according to manufacturer's protocol. Briefly, 0.5 μg of total RNA was ribo-depleted using the Ribo-Zero Gold Kit. RNA fragmentation resulted in fragments of 80-450nt, with the major peak at 160nt. First strand cDNA synthesis by random hexamers and reverse transcriptase was followed by second strand cDNA synthesis, performed in the presence of dUTP instead of dTTP. The blunt-ended double stranded cDNA was 3´adenylated and Illumina indexed adapters were ligated. The resulting library was enriched with 15 PCR cycles. The libraries were sequenced on HiSeq2000 (Illumina, Inc) in paired-end mode with a read length of 2x76bp using TruSeq SBS Kit v3-HS. Primary data analysis was carried out with the standard Illumina software Real Time Analysis (RTA 1.13.48) and followed by generation of FASTQ files.

### RNA seq data analyses and clustering

We estimated general read quality using FastQC (v0.10.1), trimmed low-quality 5′ and 3′ ends (Phred score <20) using Sickle (v1.2), merged overlapping reads (at least 10 bp) using SeqPrep (v1.1) and split merged reads with an Awk routine. Finally, paired-ends with one read shorter than 30 bp were discarded. Transcriptomic (coding RefSeq (Pruitt, 2012 #106) genes from Hg19 UCSC Table Browser [74] fixed on 2014/01) alignments were performed by TopHat2 (v2.0.1) [75]. Once aligned, we removed paired-end reads that mapped at multiple locations on transcriptome, low-quality (score <20) and discordant paired-end reads using SAMtools (v0.1.19) [76]. MarkDuplicates (PicardTools v1.99) removed PCR duplicates and Cufflinks (v2.1.1) [77] estimated gene and transcript abundances. Miscellaneous filters, statistics and plots were performed by R (v.3.1.1).

Clustering results of IMR90 together with MFH152 diploid clones 1 and 2 together and tetraploid clones 7 and 8. “Height” represents euclidean distances computed with Ward's method between whole transcriptomic profiles (in log2[FPKM]+1 unit). The more height, the more distances between profiles. “Agglomerative coefficient”, ranging from 0 to 1, represents the strength of clustering structure. The highest value, the less number of different clusters.

### Statistical procedures

Data are expressed as arithmetic means ± SEM. As indicated in the figure legends, statistical analysis was made with Graph pad Prism using ANOVA with Tukey's test as post-test. n denotes the number of different experiments or counted cell.

## SUPPLEMENTARY FIGURES AND TABLES






















