# Supplementary material for: Heterogeneity in sarcoma cell lines reveals enhanced motility of tetraploid versus diploid cells
Source: Oncotarget. 2016 Dec 27;8(10):16669–89. doi: 10.18632/oncotarget.14291 (PMC5369993; doi:10.18632/oncotarget.14291)
Supplement: Supplementary file 1 [file oncotarget-08-16669-s001.pdf]

Heterogeneity in sarcoma cell lines reveals enhanced motility of tetraploid versus diploid cells

SUPPLEMENTARY FIGURES AND TABLES

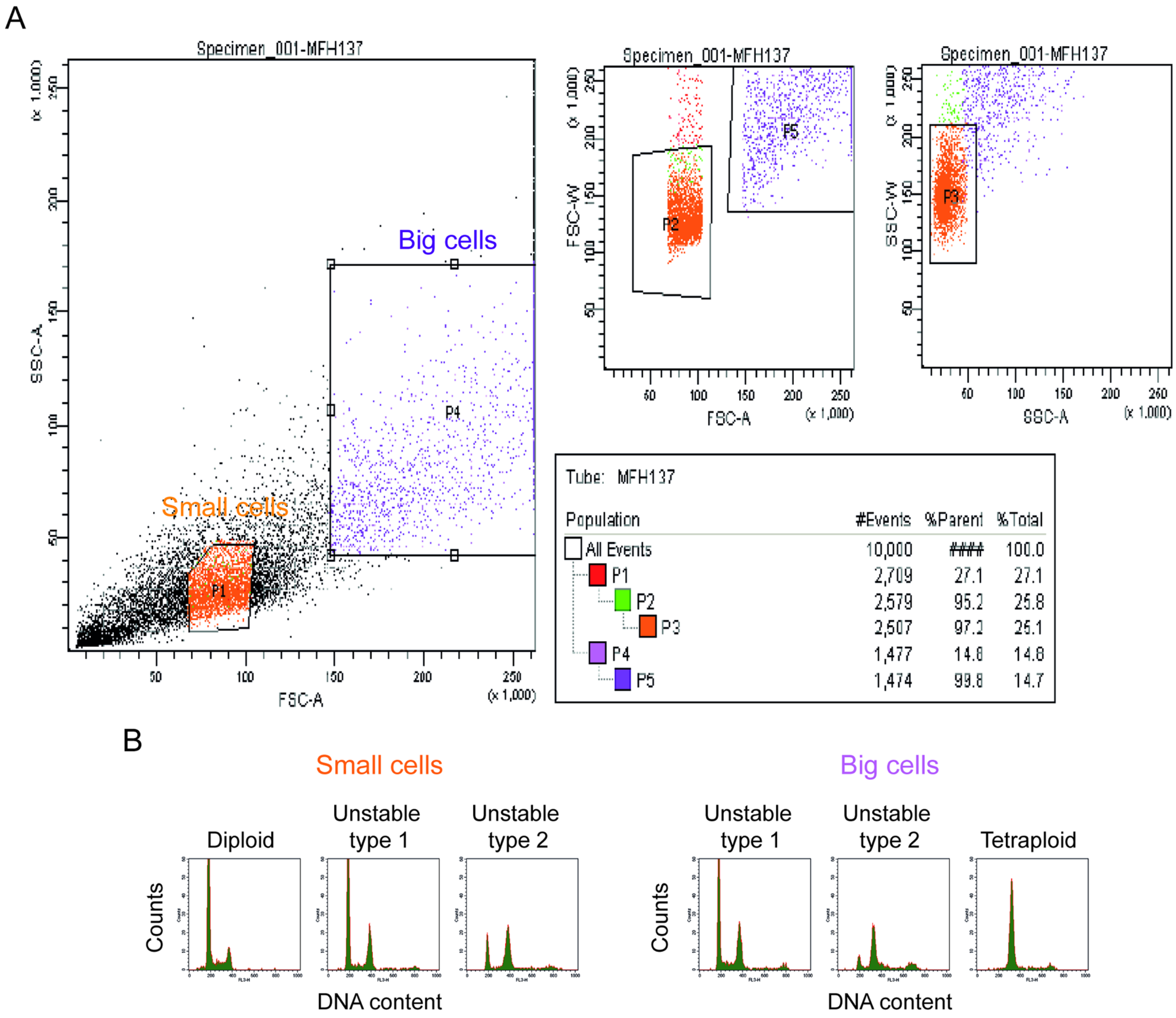

Supplementary Figure 1: Scheme of diploid and tetraploid cell sorting. (A) Separation of diploid and tetraploid cells based on size and granularity parameter using the normal light scattering parameters Forward Scatter (FSC) *vs* Side Scatter (SSC) gating. Panel **(B)** represents different cell cycle profiles of the obtained sub-clones from small *vs* big cells. Aneuploid cells with a near to diploid major G1 were named 'Unstable type 1' and those near to tetraploid major G1 'Unstable type 2'.

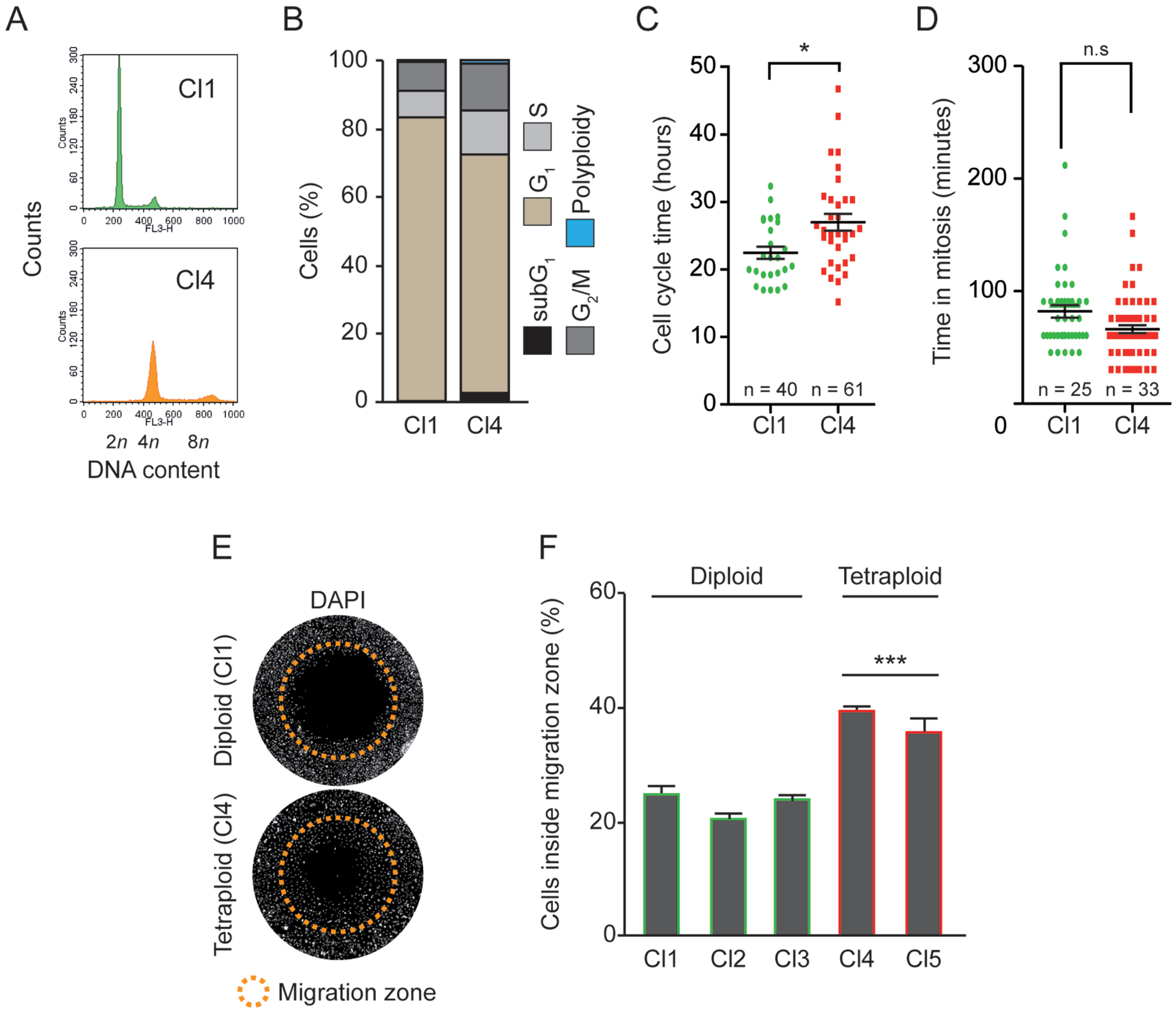

Supplementary Figure 2: MFH137 tetraploid clones are more motile than diploid clones. **(A)** and **(B)** Diploid (Cl 1) and tetraploid (Cl 4) MFH137 clones were fixed and stained with propidium iodide for the cytofluorometric assessment of cell cycle progression. Cell cycle distribution analyzed by flow cytometry is shown in panel **(A)**. Quantitative data of corresponding flow cytometry profiles are plotted in panel **(B)**. **(C)** and **(D)** Diploid (Cl 1) and tetraploid (Cl 4) MFH137 cells were imaged for up to 72h, by time-lapse microscopy, to evaluate their respective cell cycle length and time spent in mitosis. Panel **(C)** displays the quantitative data of cell cycle duration, while panel D shows the length of mitosis. *$p < 0.05$ (ANOVA test), n.s., not significant. **(E)** and **(F)** Three diploid (Cl 1-3) and two tetraploid (Cl 4-5) MFH137 cell clones were plated using the Oris™ cell migration assay and grown for 24h prior fixation. DAPI staining was used to evaluate the cells migratory potential. Representative micrographs are shown in panel E while panel F displays the percentage of nuclei (cells counting) inside the migration zone (means ± SEM; n = 3), ***$p < 0.001$ (ANOVA test, each tetraploid over each diploid)indicates significant difference between all diploid clones (one by one) comparatively to tetraploid clones (one by one). In panel **(E)**, orange circles show the migration zone at the start of the experiment (0h).

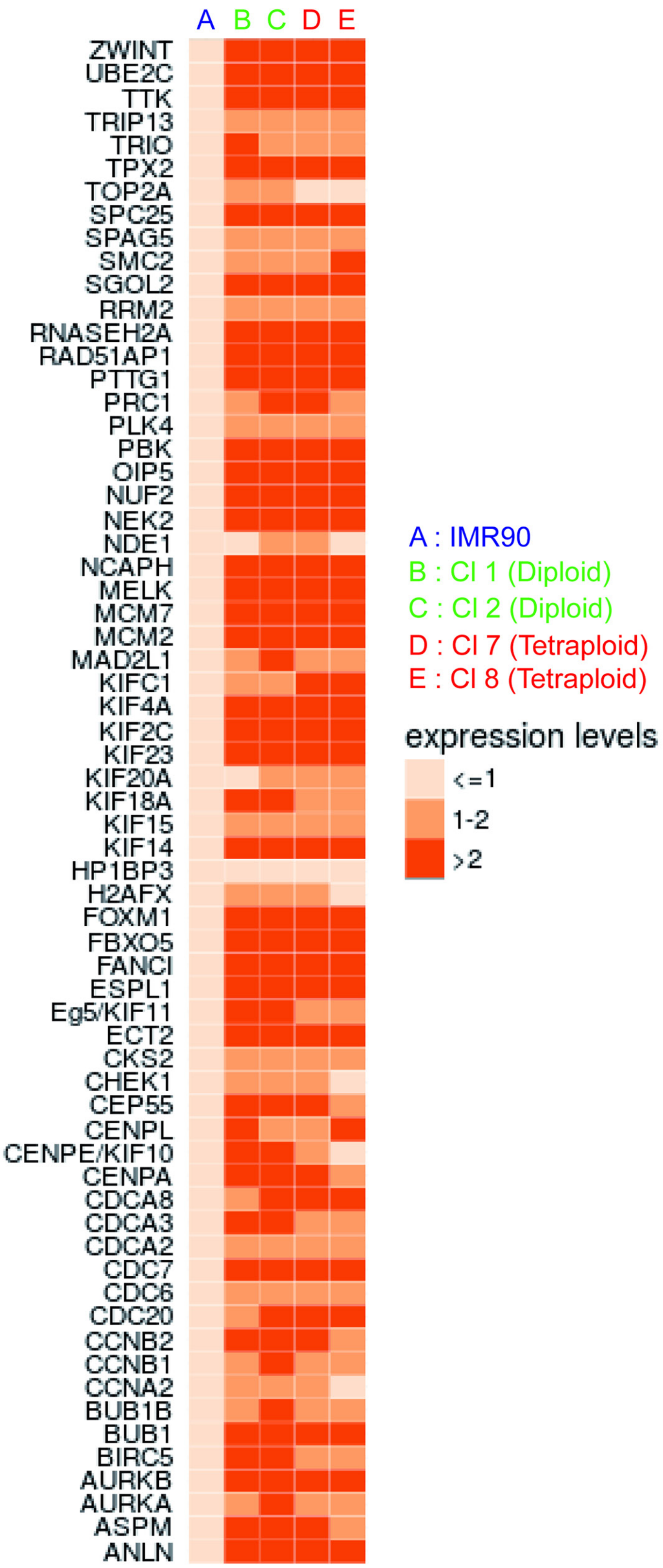

Supplementary Figure 3: Heat Map of Data presented in Table 1. CINSARC genes RNA Seq data for MFH152 diploid and tetraploid clones normalized to IMR90 expression levels. Expression levels are displayed as three classes.

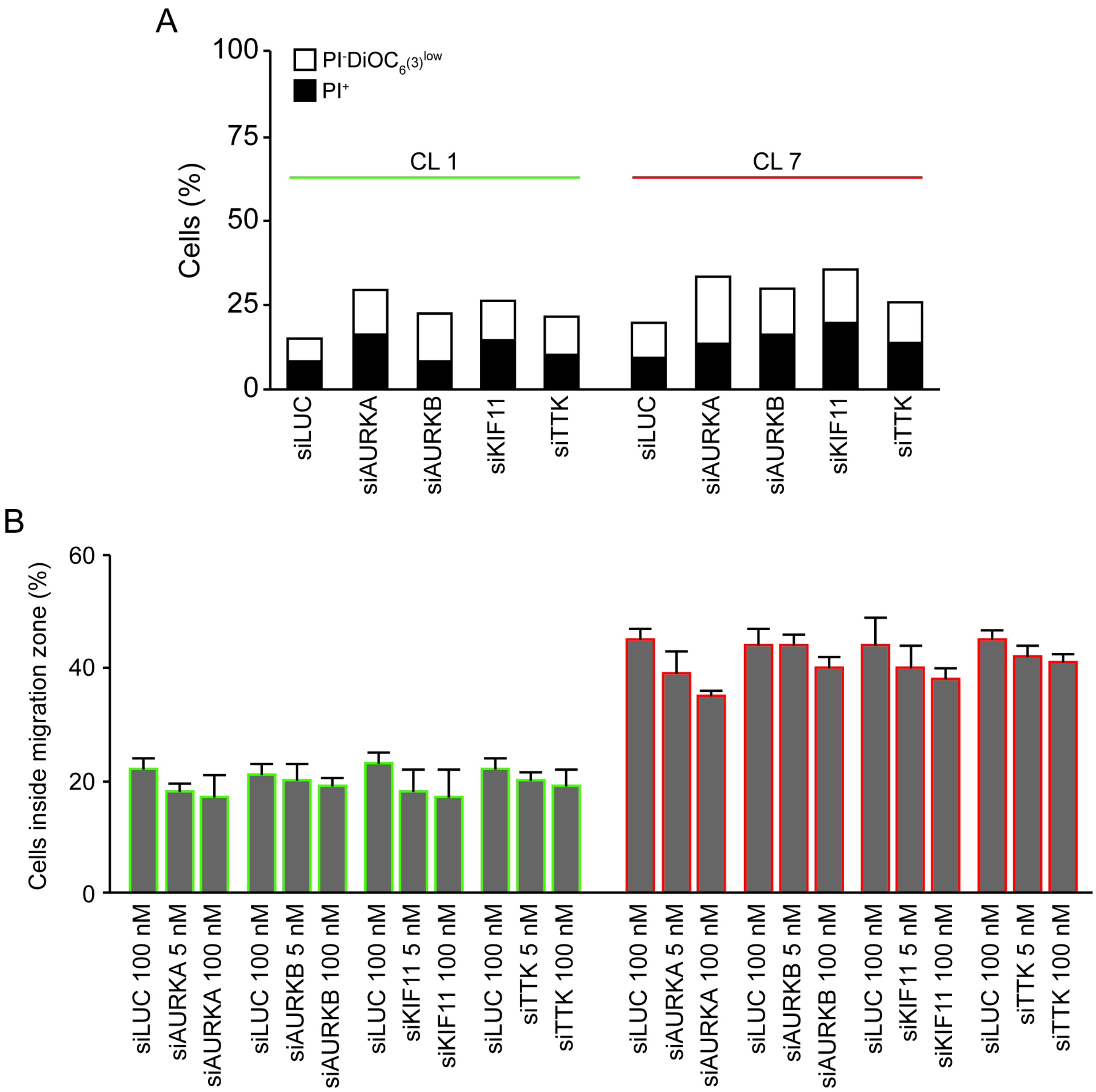

Supplementary Figure 4: siRNAs directed against either AURKA, AURKB, TTK and KIF11 have no effect on cell migration. (A) Toxicity induced in diploid (Cl1) and tetraploid (Cl7) subclones following 48h treatment with siRNAs (100nM) was assessed by flow cytometry upon co-staining with the cell death–associated parameters dyes, propidium iodide (PI) and DiOC6. **(B)** diploid (Cl 1) and tetraploid (Cl7) MFH152 clones treated for 24 hours with 5 or 100nM siRNAs were plated using the Oris™ cell migration assay. siRNA treated cells were grown for an additional 24h, fixed and stained with DAPI to measure their migratory potential. Quantitative data shows the percentage of cells (DAPI staining) inside the migration zone (means ± SEM; n=3).

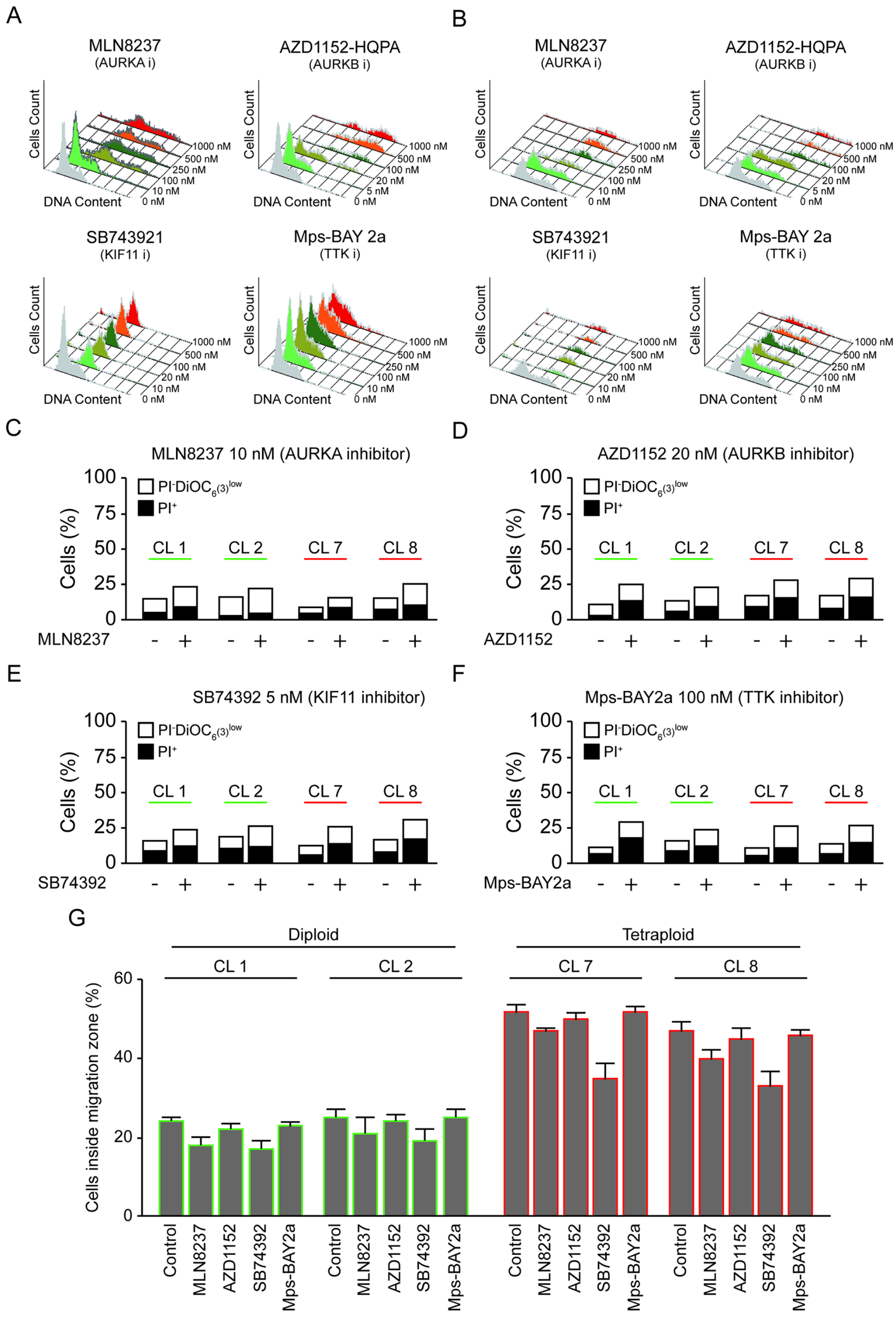

Supplementary Figure 5: highly specific drugs directed against either AURKA (MLN8237), AURKB (AZD1152-HQPA), TTK (Mps-BAy 2a) and KIF11 (SB743921) have no effect on cell migration. **(A-B)** Cell cycle distribution of diploid (Cl1) panel **(A)** and tetraploid (Cl7) panel **(B)** MFH152 clones following their exposure to increasing concentration of inhibitory drugs was analyzed by flow cytometry. **(C-F)** Toxicity induced in diploid (Cl1, Cl2) and tetraploid (Cl7, Cl8) MFH152 clones following 24h treatment drugs was assessed by flow cytometry upon co-staining with the cell death–associated parameters dyes, propidium iodide (PI) and DiOC6. **(G)** Diploid (Cl 1, Cl2) and tetraploid (Cl7, Cl8) MFH152 cells were plated using the Oris™ cell migration assay in presence of drugs (at concentrations used in C-F). After 24h growth, fixed and stained with DAPI to measure their migratory potential. Quantitative data shows the percentage of cells (DAPI staining) inside the migration zone (means ± SEM; n=3).

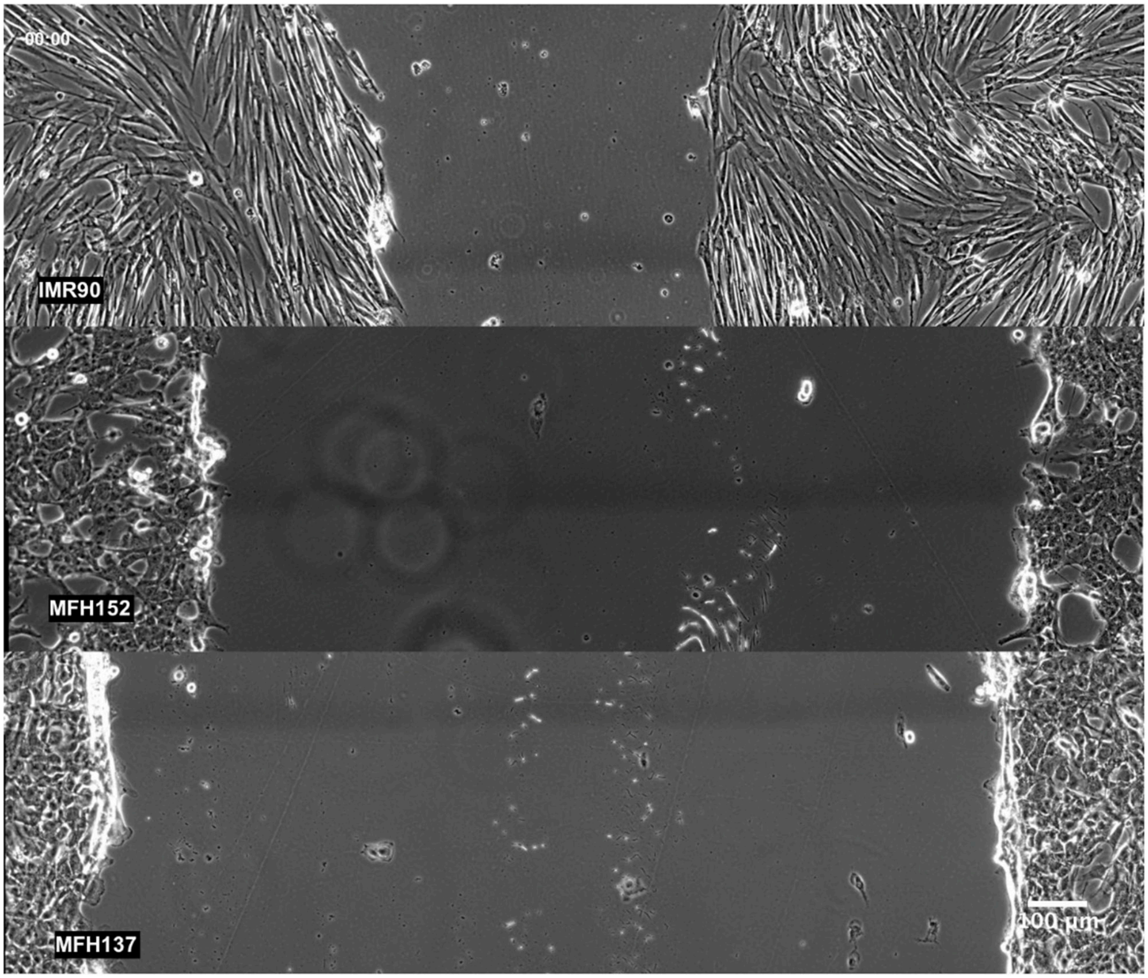

Supplementary Video 1: Wound healing assay. IMR90 human fibroblast, MFH152 and MFH137 human sarcoma cell lines were grown up to 100% confluence, and then scratched with a tip to quantify their migration potential. Images were taken every 30 minutes for 25h with phase contrast microscopy. Videos were cropped and combined to compare the three cell types.

See Supplementary Video 1

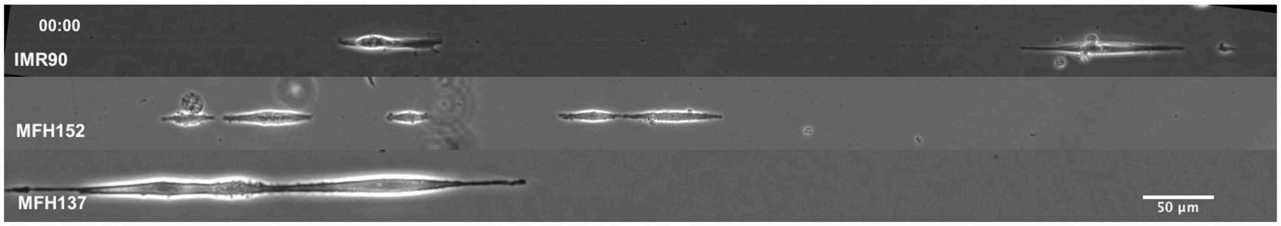

Supplementary Video 2: Cells migrations on fibronectin coated tracks. IMR90 human fibroblast, MFH152 and MFH137 human sarcoma cell lines were seeded for individual cell migration assay using the CytooChips Motility system. Each cell type was recorded in a separate well. Images were taken every 15 minutes for 34h with phase contrast microscopy. Videos were cropped and combined to show one line per cell type.

See Supplementary Video 2

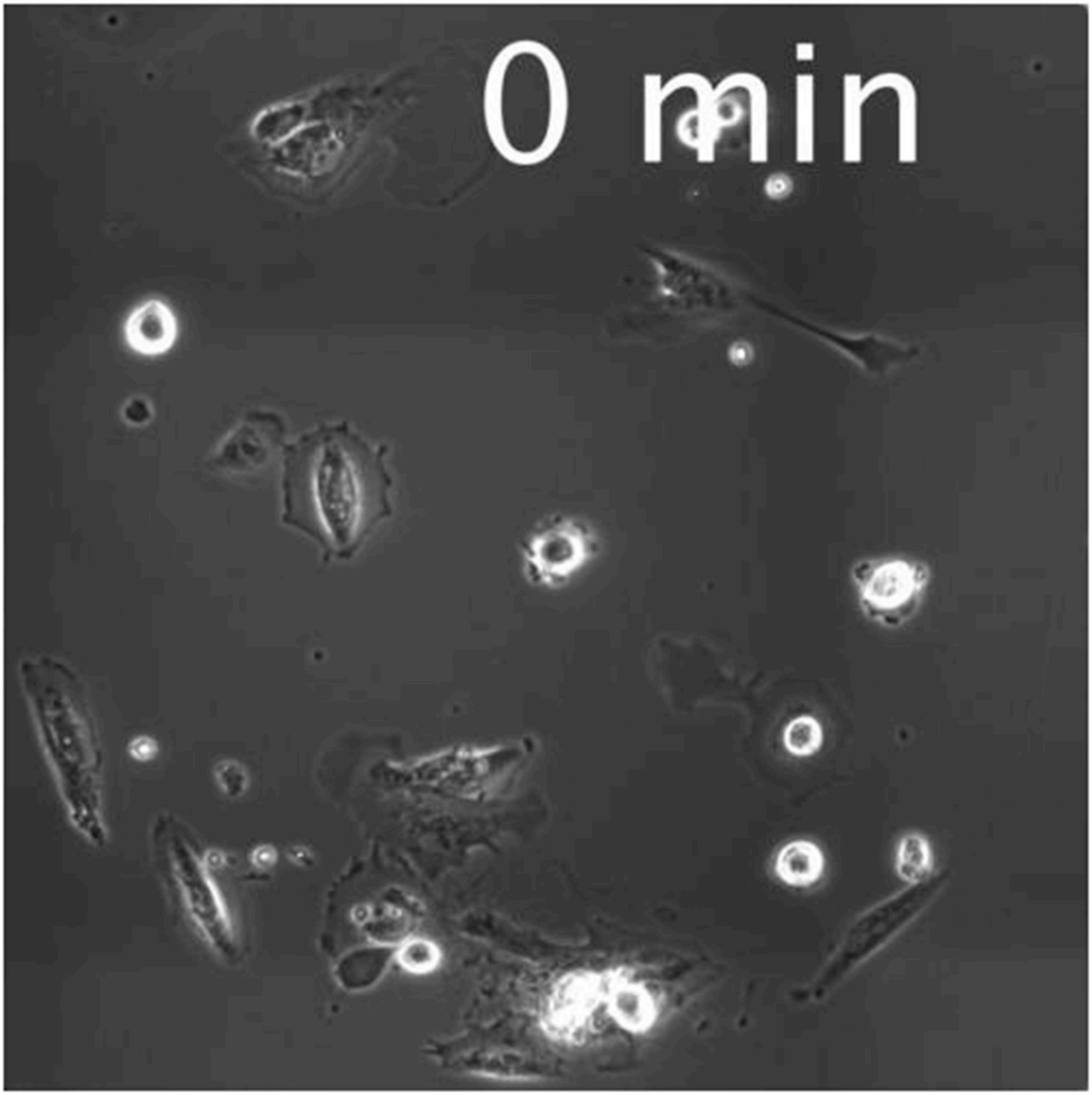

Supplementary Video 3: Analysis of cell cycle and mitosis execution. IMR90 human fibroblast, MFH152 and MFH137 human sarcoma cell lines (respectively Supplementary Videos 3-5) were seeded on fibronectin-coated slide CytooChips Arena. Images were acquired every 8 min, to quantify the length of the cell cycle and of mitosis, during 2312 minutes for IM90 and 4184 minutes for MFH152 and MFH137.

See Supplementary Video 3

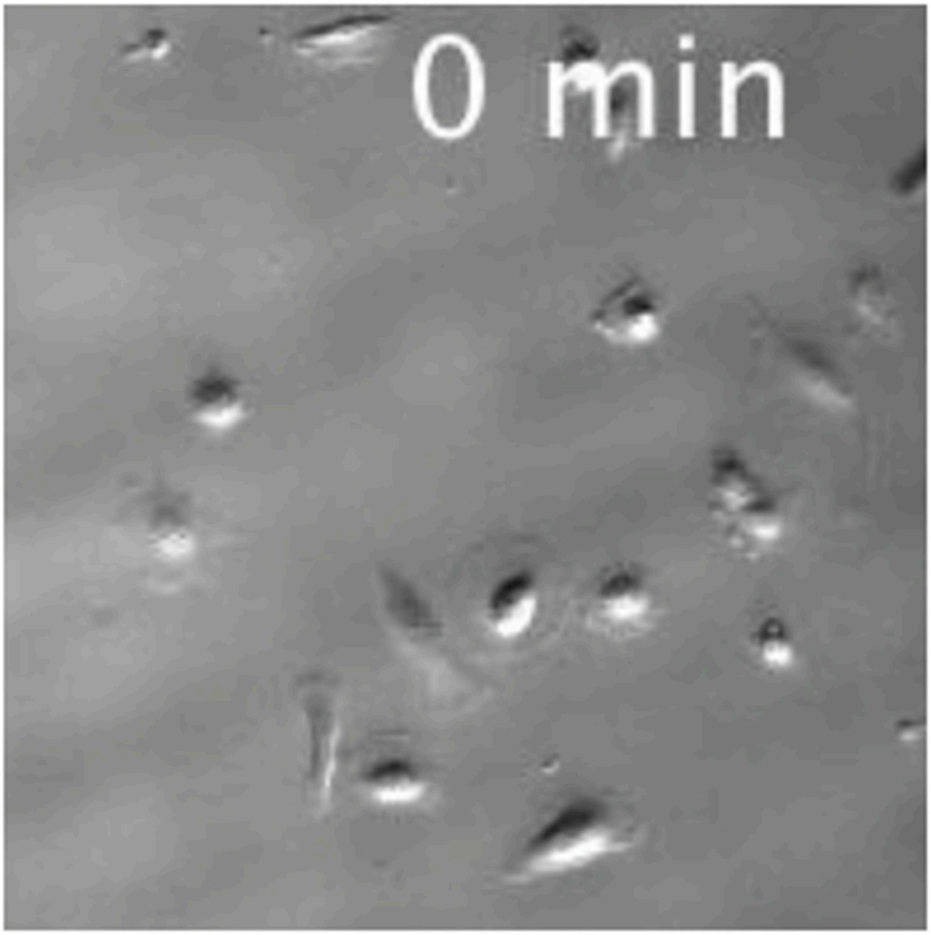

Supplementary Video 4: Analysis of cell cycle and mitosis execution. IMR90 human fibroblast, MFH152 and MFH137 human sarcoma cell lines (respectively Supplementary Videos 3-5) were seeded on fibronectin-coated slide CytooChips Arena. Images were acquired every 8 min, to quantify the length of the cell cycle and of mitosis, during 2312 minutes for IM90 and 4184 minutes for MFH152 and MFH137.

See Supplementary Video 4

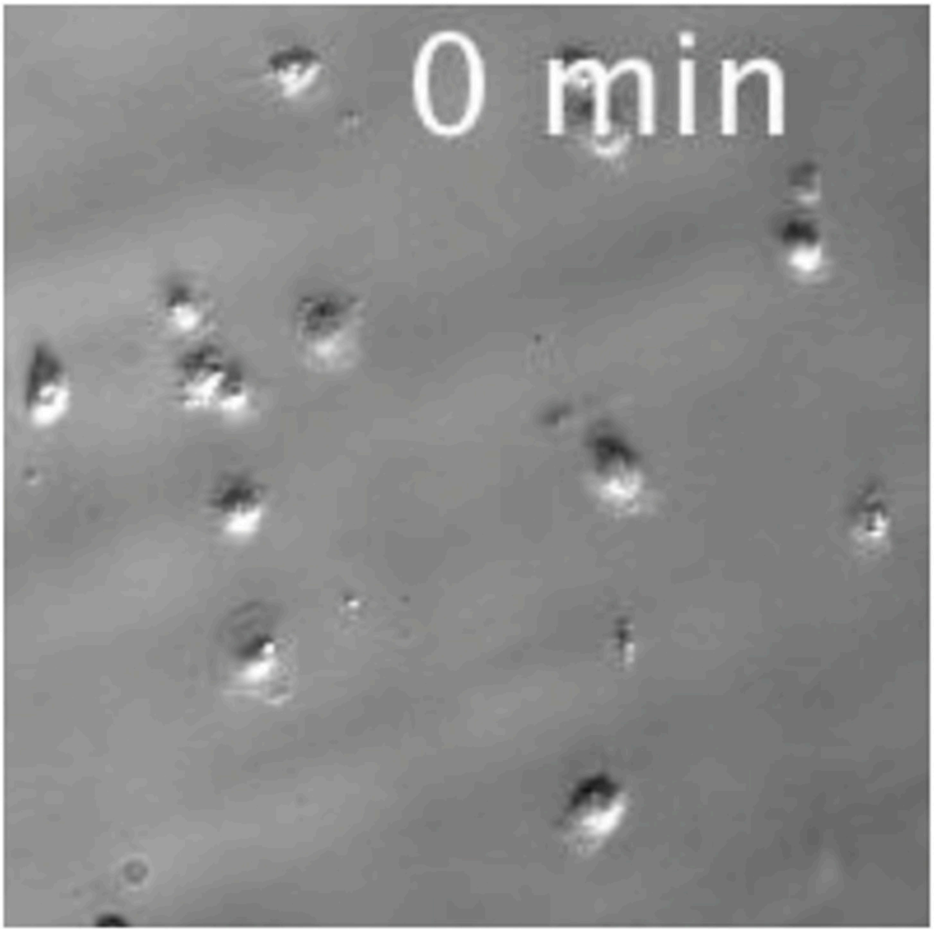

Supplementary Video 5: Analysis of cell cycle and mitosis execution. IMR90 human fibroblast, MFH152 and MFH137 human sarcoma cell lines (respectively Supplementary Videos 3-5) were seeded on fibronectin-coated slide CytooChips Arena. Images were acquired every 8 min, to quantify the length of the cell cycle and of mitosis, during 2312 minutes for IM90 and 4184 minutes for MFH152 and MFH137.

See Supplementary Video 5

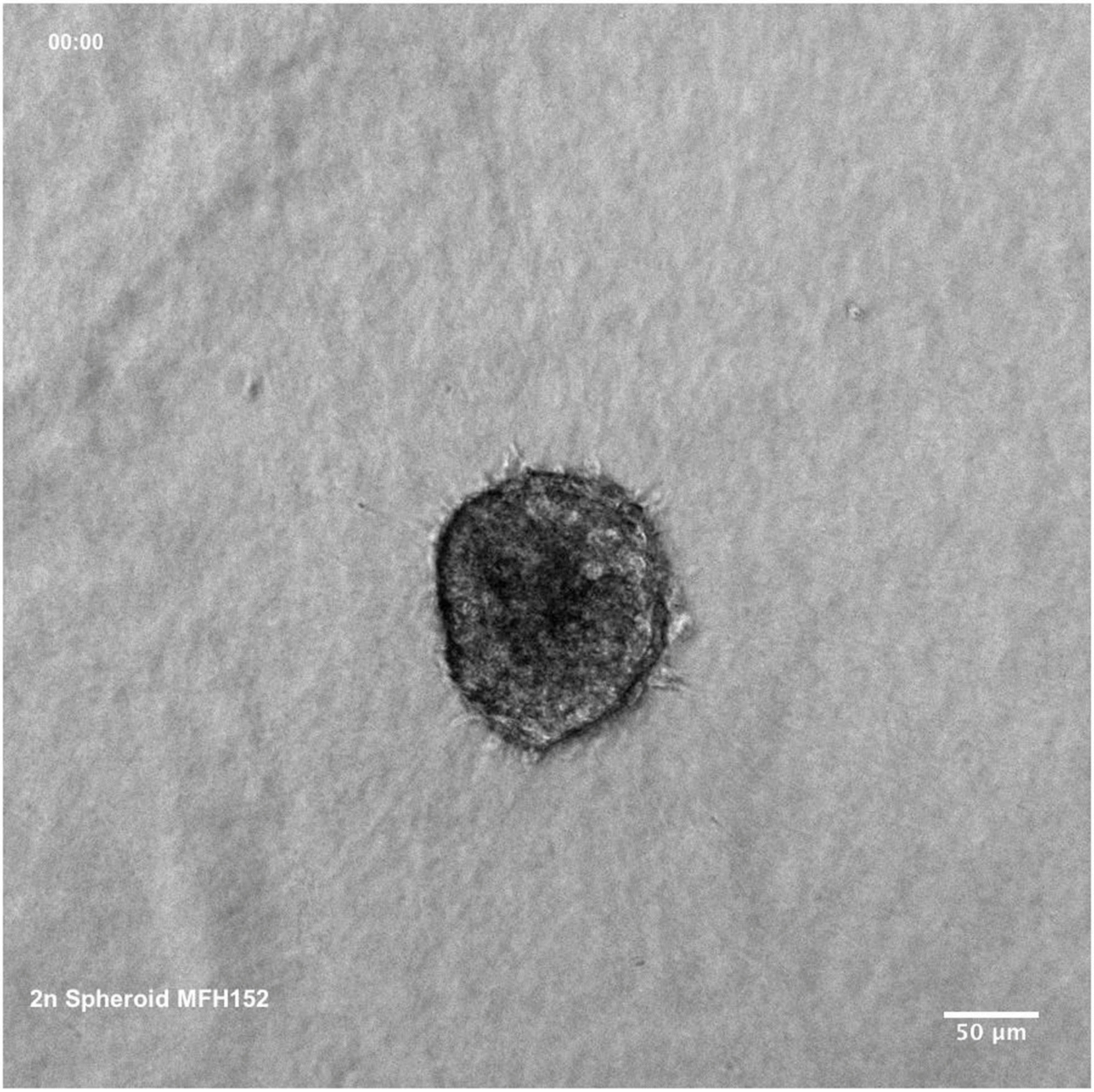

Supplementary Video 6: The 3D invasive potential of diploid MFH152 clone is inhibited by reversine and SP600125. Diploid MFH152 clone were incubated in low adherent conditions to form multicellular spheroids that were transferred to 96 wells plates containing a collagen matrix to allow invasion. Buffer (video 6) or Reversine (video 7) or SP600125 (video 8) were added to the collagen matrix and images were recorded every hour.

See Supplementary Video 6

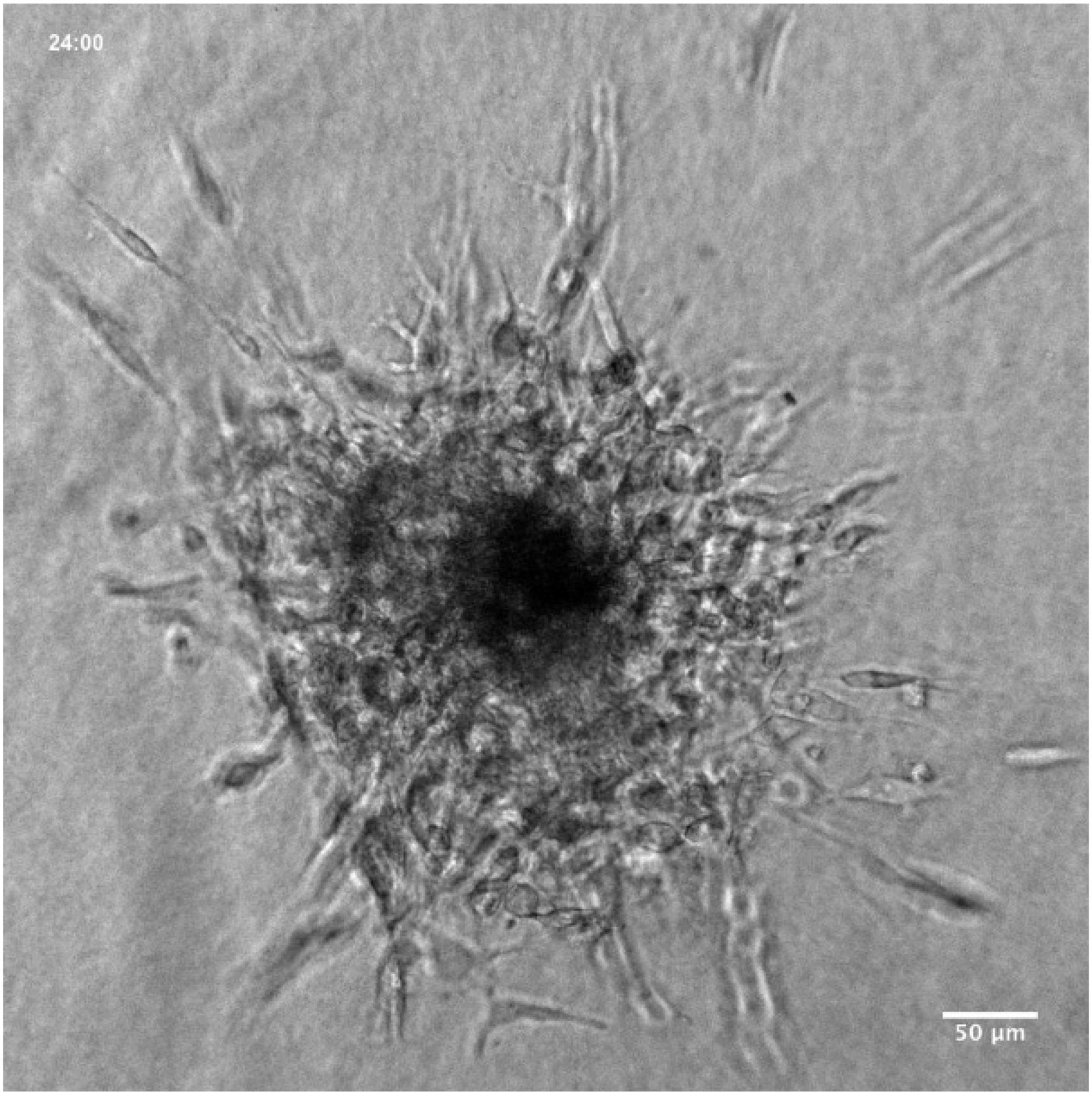

Supplementary Video 7: The 3D invasive potential of diploid MFH152 clone is inhibited by reversine and SP600125. Diploid MFH152 clone were incubated in low adherent conditions to form multicellular spheroids that were transferred to 96 wells plates containing a collagen matrix to allow invasion. Buffer (video 6) or Reversine (video 7) or SP600125 (video 8) were added to the collagen matrix and images were recorded every hour.

See Supplementary Video 7

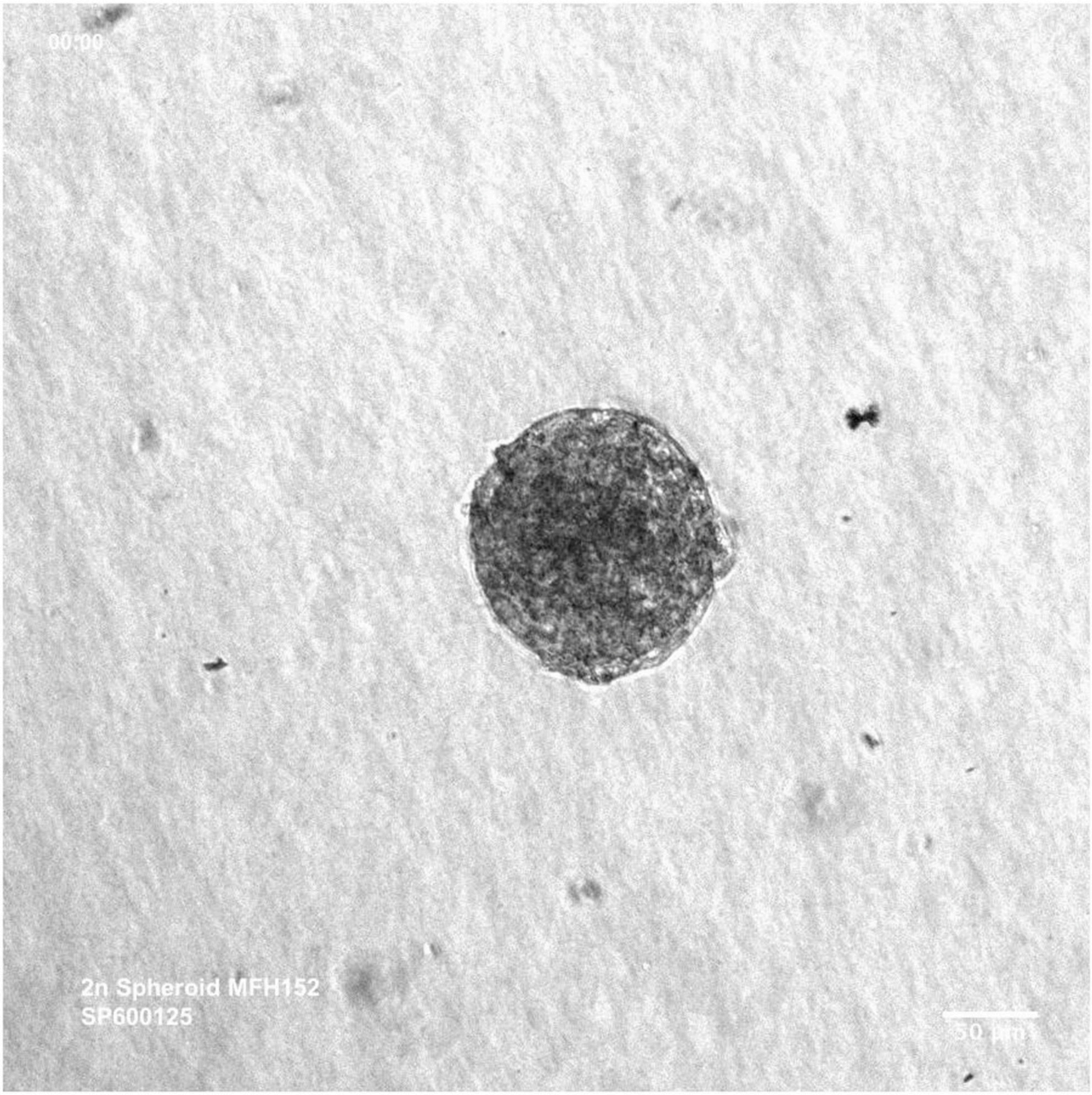

Supplementary Video 8: The 3D invasive potential of diploid MFH152 clone is inhibited by reversine and SP600125. Diploid MFH152 clone were incubated in low adherent conditions to form multicellular spheroids that were transferred to 96 wells plates containing a collagen matrix to allow invasion. Buffer (video 6) or Reversine (video 7) or SP600125 (video 8) were added to the collagen matrix and images were recorded every hour.

See Supplementary Video 8

Supplementary Table 1: Major known targets for the drugs used in this study.

See Supplementary File 1

Supplementary Table 2: Cell death ratio for MFH152 diploid and tetraploid clones upon incubation with three doses of the indicated drugs. Red labeling indicates a cell death ratio above 0.30.

See Supplementary File 2
