# Supplementary material for: Heterogeneity in sarcoma cell lines reveals enhanced motility of tetraploid versus diploid cells
Source: Oncotarget. 2016 Dec 27;8(10):16669–89. doi: 10.18632/oncotarget.14291 (PMC5369993; doi:10.18632/oncotarget.14291)
Supplement: Supplementary file 2 [file oncotarget-08-16669-s002.docx]

**Supplementary Table 1: Drug list^1^.**

| **Molecule** | **Target** | **Reference** |
| --- | --- | --- |
| AZ 3146 | TTK | ([1](#_ENREF_1)) |
| Reversine | TTK & AURKB | ([2](#_ENREF_2)) |
| SP600125 | TTK & JNK | ([3](#_ENREF_3)) |
| ZM 447439 | AURK A/B | ([4](#_ENREF_4)) |
| BI 2536 | PLK1 | ([5](#_ENREF_5)) |
| STLC | Eg5/KIF11 | (6) |
| Dimethylenastron | Eg5/KIF11 | (7) |
| GSK 923295 | CENPE | (8) |
| SB203508 | p38 | (9) |
| RO 3306 | CDK1 | (10) |
| Cdk1 Inhibitor III | CDK1 | (11) |
| Roscovitine | CDK1 | (12) |
| NSC 95397 | CDC25 | (13) |
| IPA3 | PAK1 | (14) |
| Y-27632 | ROCK | (15) |
| ITX-3 | Trio | (16) |
| Nocodazole | MT Polymerisation | (17) |
| Paclitaxel/Taxol | MT dynamics | (18) |
| Blebbistatin | Myosin II | (19) |
| Cytochalasin B | Actine | (20) |
| MG 132 | Proteasome | (21) |
| Velcade | Proteasome | (22) |
| MLN 8237 | AURKA | (23) |
| AZD1152-HQPA | AURKB | (24) |
| SB743921 | KIF11 | (25) |
| Mps-BAY2A | TTK | (26) |

1 The drugs used for the screen are labled in red

**Reference.**

1. Hewitt L*, et al.* (2010) Sustained Mps1 activity is required in mitosis to recruit O-Mad2 to the Mad1-C-Mad2 core complex. (Translated from eng) *J Cell Biol* 190(1):25-34 (in eng).

2. Santaguida S, Tighe A, D'Alise AM, Taylor SS, & Musacchio A (2010) Dissecting the role of MPS1 in chromosome biorientation and the spindle checkpoint through the small molecule inhibitor reversine. (Translated from eng) *J Cell Biol* 190(1):73-87 (in eng).

3. Schmidt M, Budirahardja Y, Klompmaker R, & Medema RH (2005) Ablation of the spindle assembly checkpoint by a compound targeting Mps1. (Translated from eng) *EMBO Rep* 6(9):866-872 (in eng).

4. Girdler F*, et al.* (2006) Validating Aurora B as an anti-cancer drug target. (Translated from eng) *J Cell Sci* 119(Pt 17):3664-3675 (in eng).

5. Steegmaier M*, et al.* (2007) BI 2536, a potent and selective inhibitor of polo-like kinase 1, inhibits tumor growth in vivo. (Translated from eng) *Curr Biol* 17(4):316-322 (in eng).

6. Debonis S*, et al.* (2008) Structure-activity relationship of S-trityl-L-cysteine analogues as inhibitors of the human mitotic kinesin Eg5. (Translated from eng) *J Med Chem* 51(5):1115-1125 (in eng).

7. Liu M*, et al.* (2008) Validating the mitotic kinesin Eg5 as a therapeutic target in pancreatic cancer cells and tumor xenografts using a specific inhibitor. (Translated from eng) *Biochem Pharmacol* 76(2):169-178 (in eng).

8. Qian X*, et al.* (2010) Discovery of the First Potent and Selective Inhibitor of Centromere-Associated Protein E: GSK923295. (Translated from eng) *ACS Med Chem Lett* 1(1):30-34 (in eng).

9. Underwood DC*, et al.* (2000) SB 239063, a potent p38 MAP kinase inhibitor, reduces inflammatory cytokine production, airways eosinophil infiltration, and persistence. (Translated from eng) *J Pharmacol Exp Ther* 293(1):281-288 (in eng).

10. Vassilev LT*, et al.* (2006) Selective small-molecule inhibitor reveals critical mitotic functions of human CDK1. (Translated from eng) *Proc Natl Acad Sci U S A* 103(28):10660-10665 (in eng).

11. Brachwitz K*, et al.* (2003) Evaluation of the first cytostatically active 1-aza-9-oxafluorenes as novel selective CDK1 inhibitors with P-glycoprotein modulating properties. (Translated from eng) *J Med Chem* 46(5):876-879 (in eng).

12. De Azevedo WF*, et al.* (1997) Inhibition of cyclin-dependent kinases by purine analogues: crystal structure of human cdk2 complexed with roscovitine. (Translated from eng) *Eur J Biochem* 243(1-2):518-526 (in eng).

13. Ducruet AP*, et al.* (2000) Identification of new Cdc25 dual specificity phosphatase inhibitors in a targeted small molecule array. (Translated from eng) *Bioorg Med Chem* 8(6):1451-1466 (in eng).

14. Wang Z, Oh E, Clapp DW, Chernoff J, & Thurmond DC (2011) Inhibition or ablation of p21-activated kinase (PAK1) disrupts glucose homeostatic mechanisms in vivo. (Translated from eng) *J Biol Chem* 286(48):41359-41367 (in eng).

15. Narumiya S, Ishizaki T, & Uehata M (2000) Use and properties of ROCK-specific inhibitor Y-27632. (Translated from eng) *Methods Enzymol* 325:273-284 (in eng).

16. Bouquier N*, et al.* (2009) A cell active chemical GEF inhibitor selectively targets the Trio/RhoG/Rac1 signaling pathway. (Translated from eng) *Chem Biol* 16(6):657-666 (in eng).

17. Eilers U, Klumperman J, & Hauri HP (1989) Nocodazole, a microtubule-active drug, interferes with apical protein delivery in cultured intestinal epithelial cells (Caco-2). (Translated from eng) *J Cell Biol* 108(1):13-22 (in eng).

18. Terzis AJ*, et al.* (1997) Proliferation, migration and invasion of human glioma cells exposed to paclitaxel (Taxol) in vitro. (Translated from eng) *Br J Cancer* 75(12):1744-1752 (in eng).

19. Straight AF*, et al.* (2003) Dissecting temporal and spatial control of cytokinesis with a myosin II Inhibitor. (Translated from eng) *Science* 299(5613):1743-1747 (in eng).

20. Haidle AM & Myers AG (2004) An enantioselective, modular, and general route to the cytochalasins: synthesis of L-696,474 and cytochalasin B. (Translated from eng) *Proc Natl Acad Sci U S A* 101(33):12048-12053 (in eng).

21. Yan XB*, et al.* (2007) Caspase-8 dependent osteosarcoma cell apoptosis induced by proteasome inhibitor MG132. (Translated from eng) *Cell Biol Int* 31(10):1136-1143 (in eng).

22. Adams J*, et al.* (1999) Proteasome inhibitors: a novel class of potent and effective antitumor agents. (Translated from eng) *Cancer Res* 59(11):2615-2622 (in eng).

23. [Manfredi MG](https://www.ncbi.nlm.nih.gov/pubmed/?term=Manfredi%20MG%5BAuthor%5D&cauthor=true&cauthor_uid=22016509), *et al* (2011) Characterization of Alisertib (MLN8237), an investigational small-molecule inhibitor of aurora A kinase using novel in vivo pharmacodynamic assays. *Clin Cancer Res.* 17(24):7614-24

24. Löwenberg B *et al,* (2011) Phase 1/2 study to assess the safety, efficacy, and pharmacokinetics of barasertib (AZD1152) in patients with advanced acute myeloid leukemia. *Blood* 118:6030-6036

25. Holen KD *et al* (2011) A first in human study of SB-743921, a kinesin spindle protein inhibitor, to determine pharmacokinetics, biologic effects and establish a recommended phase II dose. *Cancer Chemother Pharmacol*. 67(2):447-54

26. Jemaà *et al* (2013) Characterization of novel MPS1 inhibitors with preclinical anticancer activity. *Cell Death Differ.* 20:1532-45.
