# Supplementary material for: Heterogeneity in sarcoma cell lines reveals enhanced motility of tetraploid versus diploid cells
Source: Oncotarget. 2016 Dec 27;8(10):16669–89. doi: 10.18632/oncotarget.14291 (PMC5369993; doi:10.18632/oncotarget.14291)
Supplement: Supplementary file 3 [file oncotarget-08-16669-s003.docx]

**Supplementary Table 2^[[1]](#footnote-1)^: Cell death ratio for MFH152 diploid and tetraploid clones upon incubation with three doses of the indicated drugs. Red labeling indicates a cell death ratio above 0.30.**

|  |  | **Cell Death** | | | | | |
| --- | --- | --- | --- | --- | --- | --- | --- |
|  |  | **0.1 μM** | | **1 μM** | | **10 μM** | |
|  |  | **Diploid** | **Tetraploid** | **Diploid** | **Tetraploid** | **Diploid** | **Tetraploid** |
| 1 | **AZ 3146** | 0.10 | 0.00 | 0.24 | 0.00 | **0.37** | 0.23 |
| 2 | **Reversine** | 0.15 | 0.00 | 0.18 | 0.12 | 0.20 | 0.20 |
| 3 | **SP600125** | 0.12 | 0.00 | 0.19 | 0.00 | 0.21 | 0.11 |
| 4 | **ZM 447439** | 0.05 | 0.00 | **0.32** | 0.24 | **0.52** | **0.52** |
| 5 | **BI 2536** | **0.56** | **0.38** | **0.74** | **0.48** | **0.79** | **0.87** |
| 6 | **STLC** | **0.67** | 0.19 | **0.84** | **0.59** | **0.84** | **0.70** |
| 7 | **Dimethylenastron** | 0.13 | 0.07 | **0.87** | **0.48** | **0.90** | **0.65** |
| 8 | **GSK 923295** | 0.05 | 0.08 | **0.50** | **0.34** | **0.70** | **0.39** |
| 9 | **SB203508** | 0.04 | 0.02 | 0.00 | 0.00 | 0.00 | 0.00 |
| 10 | **RO 3306** | 0.12 | 0.16 | 0.08 | 0.00 | **0.77** | **0.59** |
| 11 | **Cdk1 Inhibitor III** | 0.00 | 0.23 | 0.01 | 0.19 | **0.84** | **0.65** |
| 12 | **Roscovitine** | 0.17 | 0.07 | 0.14 | **0.52** | **0.50** | **0.28** |
| 13 | **NSC 95397** | 0.02 | 0.11 | 0.00 | 0.06 | **0.98** | **0.97** |
| 14 | **IPA3** | 0.00 | 0.00 | 0.00 | 0.00 | 0.00 | 0.00 |
| 15 | **Y-27632** | 0.02 | 0.00 | 0.06 | 0.00 | 0.02 | 0.03 |
| 16 | **ITX-3** | 0.00 | 0.00 | 0.00 | 0.03 | 0.00 | 0.00 |
| 17 | **Nocodazole** | **0.34** | **0.59** | **0.46** | **0.63** | **0.58** | **0.74** |
| 18 | **Paclitaxel/Taxol** | 0.22 | **0.32** | **0.72** | **0.70** | **0.77** | **0.74** |
| 19 | **Blebbistatin** | 0.01 | 0.04 | 0.11 | 0.06 | 0.10 | 0.06 |
| 20 | **Cytochalasin B** | 0.19 | 0.08 | **0.31** | **0.44** | **0.81** | **0.64** |
| 21 | **MG 132** | 0.00 | 0.00 | **0.41** | **0.30** | **0.62** | **0.52** |
| 22 | **Velcade** | **0.53** | **0.35** | **0.60** | **0.41** | **0.68** | **0.54** |

1. Measures of cell death induced by the different drugs, were performed in quadruplate and in four independent experiments. Basically, following 24 hours cell treatment in presence or absence of drugs, all nuclei, were counted in a 2.8 mm^2^ circle surrounding and including the migration zone. The cell death ratio presented in the table represents the ratio of counted nuclei in treated versus untreated cells. [↑](#footnote-ref-1)
